# Carbon compensation or fuel displacement? Biomass allocation trade-offs for decarbonizing aviation

**DOI:** 10.1016/j.isci.2026.116848

**Published:** 2026-07-16

**Authors:** Hassan El-Houjeiri, Adam R. Brandt, Mohammad S. Masnadi

**Affiliations:** 1 Department of Climate and Sustainability, KAPSARC, Riyadh, Saudi Arabia; 2 Stanford Doerr School of Sustainability, Stanford University, Stanford, CA, USA; 3 Department of Chemical and Petroleum Engineering, University of Pittsburgh, Pittsburgh, PA, USA

**Keywords:** biochar, sustainable aviation fuel, SAF, waste biomass, techno-economic analysis, carbon compensation, abatement cost, life cycle assessment

## Abstract

Waste biomass can deliver climate mitigation through two fundamentally different routes: durable carbon removal via biochar and fossil-jet displacement via sustainable aviation fuel (SAF). We harmonize published techno-economic assessments to a common 2024 delivered-to-market basis and compare both pathways as climate-service abatement cost (USD ${\text{tCO}}_{2}^{-1}$ removed or avoided). For biochar, we combine an empirical cost-capacity relationship with audited net-removal factors. For SAF, we distinguish the commercially dominant waste-lipid route from the waste- and residue-derived pathways needed for larger long-run scale. Commercial biochar systems currently deliver a median abatement cost of about 177 USD ${\text{tCO}}_{2}^{-1}$ (5th–95th percentile: 111–497 USD ${\text{tCO}}_{2}^{-1}$), while waste-derived SAF centers near 448 USD ${\text{tCO}}_{2}^{-1}$. Current SAF supply is still overwhelmingly lipid-based, whereas the lignocellulosic and municipal-solid-waste routes needed for larger long-term aviation abatement remain earlier in commercialization. These differences suggest complementary timelines: biochar is a near-term removal option, while SAF remains a longer-term aviation decarbonization pathway.

## Highlights

• Biochar removal and waste-derived SAF harmonized on a 2024 $/tCO2 climate basis
• Biochar abates CO2 near 177 $/t versus about 448 $/t for waste-derived SAF
• Biochar is mature today; most waste-derived SAF routes are still precommercial
• Result is a temporal complement, not a fixed biomass-allocation rule

## Graphical abstract

**Figure:**
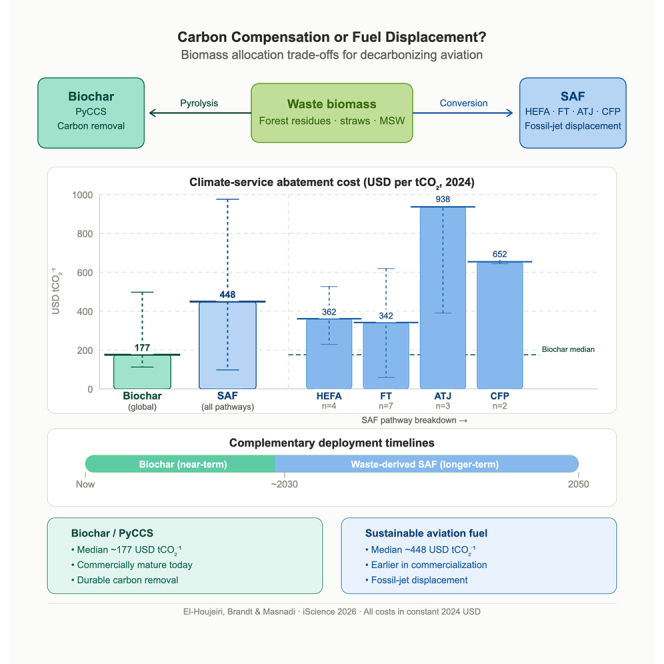

## Teaser

Environmental science; Environmental analysis

## Introduction

### A feasibility comparison of biochar and SAF

Waste biomass can support substantial climate mitigation, yet the pathways through which it delivers this mitigation differ markedly in mechanism, maturity, and techno-economic performance. Biomass presents intrinsic constraints—including high oxygen content, low energy density, and spatially fragmented supply chains—that complicate large-scale deployment. Consequently, its strategic mitigation value lies primarily in hard-to-abate sectors where low-carbon alternatives are limited, such as aviation decarbonization. Prioritizing the most carbon-efficient and economically viable conversion pathways is therefore essential to scale biomass utilization effectively for climate mitigation. Two prominent approaches—pyrogenic carbon capture and storage (PyCCS), which converts biomass into stable biochar, and the production of sustainable aviation fuel (SAF) from waste feedstocks—serve distinct roles within the broader mitigation portfolio. PyCCS provides durable atmospheric carbon removal by stabilizing biogenic carbon in a solid form,^1^^,^^2^ whereas SAF pathways convert biomass into liquid fuels that displace fossil jet fuel and thereby avoid new fossil CO_2_ emissions. These fundamentally different mechanisms—carbon removal versus fossil-carbon displacement—imply distinct sectoral roles, timelines, and carbon-market valuation logics.

Despite rapid development in both domains, there is no harmonized, data-driven comparison of the cost at which waste biomass can be transformed into verified climate mitigation through either pathway—a gap that limits clear signals for carbon markets and deployment decisions. Published techno-economic assessments (TEAs) for biochar production span a wide range—from 112 to 3,335 USD t^−1^—reflecting variation in feedstock, technology archetype, scale, coproduct handling, and system boundaries^3^^,^^4^^,^^5^^,^^6^^,^^7^^,^^8^^,^^9^^,^^10^^,^^11^^,^^12^ (see Section S1; Tables S1–S4). Waste-based SAF pathways exhibit similarly broad abatement-cost ranges of roughly 100–1,000 USD t^−1^CO_2_ depending on feedstock and conversion route (see Section S5; Table S15). Without a consistent framework for comparing these heterogeneous results, it remains difficult to assess the relative feasibility of these pathways or to understand how they may jointly contribute to climate goals.

Commercial SAF discussions often separate three feedstock classes that differ sharply in maturity and scale: waste fats, oils, and greases converted via hydroprocessed esters and fatty acids (HEFAs); sugar- or starch-derived alcohol routes; and lignocellulosic or municipal-solid-waste routes converted through Fischer–Tropsch (FT), alcohol-to-jet (ATJ), or catalytic fast pyrolysis (CFP). Current global SAF production remains small—about 2 Mt in 2025, or roughly 0.7% of jet-fuel demand—and HEFA is still the only commercially scaled pathway, whereas most lignocellulosic and waste-based routes remain earlier in commercialization. In InternatioInternational Air Transport Association's (nal Air Transport Association's (IATA) core 2050 forecast, oil-based HEFA supplies just over 15% of SAF, while agroforestry residues contribute about one-quarter and municipal solid waste about 7%, underscoring that large-scale aviation abatement cannot rely on waste lipids alone.^13^

In this study, we synthesize TEA data for both pathways and harmonize them to a common 2024 USD delivered-to-market basis. We pair these harmonized costs with carbon-removal factors from audited PyCCS projects and life cycle carbon intensities from recent SAF studies to estimate the cost of delivering a verified tonne of CO_2_ mitigation. For biochar, the cost numerator is the delivered minimum selling price in 2024 USD per tonne of dry biochar, and the carbon denominator is the audited net-removal factor (t CO_2_ removed per tonne of biochar). For SAF, the numerator is the fuel-cost premium relative to fossil jet fuel, and the carbon denominator is the life cycle carbon-intensity difference relative to the fossil baseline, consistent with Carbon Offsetting and Reduction Scheme for International Aviation (CORSIA) eligible-fuel accounting conventions. This keeps removal and avoidance accounting distinct while expressing both pathways on a common climate-service basis of USD per tonne of CO_2_ removed or avoided (Table 1).^14^^,^^15^

**Table 1:** Comparison framework used to harmonize biochar and waste-to-SAF abatement costs

Aspect	Biochar/PyCCS	Waste-derived SAF
Climate service	durable carbon removal	fossil-jet displacement (avoidance)
Cost numerator	delivered minimum selling price in 2024 USD per tonne of dry biochar	Fuel cost premium relative to fossil jet fuel, expressed in 2024 USD
Carbon denominator	audited net removal factor from issued CO_2_ Removal Certificates (CORCs), in t CO_2_ removed per tonne of dry biochar	life cycle carbon-intensity reduction relative to fossil jet, in t CO_2_ avoided per unit fuel
Dataset used	17 harmonized biochar TEA observations from 10 studies; 8 standalone slow-pyrolysis cases retained for the core scaling fit; 19 audited CORC factors for net removal	16 harmonized waste/residue-derived SAF cases spanning HEFA, FT, ATJ, and CFP
Included feedstocks	forest residues, orchard prunings, straw, sludge, digestate, manure, olive residue, empty fruit bunch, and related wastes/residues	waste oils and fats, municipal solid waste, agricultural residues, woody residues, and related wastes/residues; crop-based starch routes excluded from the envelope
Co-product treatment	preserved as reported in source TEAs; cases with credits >30% of gross cost excluded from the core scaling fit to focus on intrinsic production economics	preserved as reported in source studies; no further normalization because public pathway-specific data remain sparse
Boundary and certification	delivered-to-market cost for biochar; net-removal denominator already deducts upstream emissions through audited registry accounting	delivered fuel cost and pathway-specific life cycle carbon intensity relative to fossil jet under CORSIA-style accounting conventions
Financing assumptions	source-study discount rates, plant lifetimes, and financing conventions retained; not re-underwritten across studies	source-study discount rates, plant lifetimes, and financing conventions retained; not re-underwritten across studies
Uncertainty treatment	empirical cost–capacity fit with Student-t confidence bounds, Monte Carlo propagation, and direct sampling of audited CORC factors	cross-technology empirical envelope fit to the compiled pathway values; median is descriptive of the literature sample, not a forecast for one pathway

Facility-level feedstocks, system boundaries, and harmonized values are reported in Tables S1–S4 for biochar and Table S15 for SAF to allow direct tracing of the exact cases entering the comparison.

This feasibility comparison clarifies the different temporal advantages of the pathways. Biochar systems enable near-term mobilization of waste biomass into durable carbon removals. They are technologically mature, already deployed commercially across a wide range of scales, and have recently gained traction with several major technology companies initiating multi-year procurement of biochar-based removal credits to complement their net-zero strategies. Waste-to-SAF pathways, while essential for decarbonizing aviation, remain comparatively energy-intensive and costlier at present, with several conversion routes still progressing toward large-scale commercial readiness. Our analysis therefore does not rank or prescribe between pathways; rather, it contextualizes how their climate-service potential emerges under current techno-economic constraints and stages of maturity. The comparison should not be read as a normative biomass-allocation rule: actual allocation should depend on local feedstock availability, competing uses, policy incentives, infrastructure, and sector-specific decarbonization priorities.

This comparison necessarily reflects differences in data availability and commercial maturity between the two pathways. For biochar, a growing body of plant-level techno-economic studies and audited carbon-removal projects enables empirical cost-capacity fitting and uncertainty propagation. For SAF, the harmonized envelope intentionally includes waste-lipid HEFA together with waste- and residue-derived FT, ATJ, and CFP cases, but excludes crop-based starch routes to maintain a waste-biomass comparison. As a result, SAF abatement costs are synthesized here as a cross-technology envelope rather than resolved through pathway-specific scaling relationships. The aggregate SAF distribution is therefore not a pathway-specific learning curve or deployment forecast; it is a harmonized literature envelope across HEFA, FT, ATJ, and CFP cases. This asymmetry reflects current evidence rather than analytical preference and highlights a priority area for future research as SAF technologies mature and deployment expands.

The sections that follow develop an empirical cost-capacity scaling relationship for biochar production (see Section S1.4.4), examine how variability in carbon removal influences abatement cost (Sections S3 and S4), and compare these results with harmonized abatement-cost distributions for waste-based SAF (Section S5). These insights provide a data-driven foundation for understanding the complementary climate roles of biochar and SAF and identify open questions for optimizing the use of waste biomass in climate mitigation.

## Results

### Economics of biochar production

Across the global landscape of biochar production, technology diversity and operational scale drive a remarkably wide cost range. TEAs conducted over the past decade encompass continuous slow-pyrolysis systems, mobile fire-control units, hydrothermal carbonization with post-carbonization, microwave-assisted pyrolysis, and combined-heat-and-power configurations (Section S1.2). Among these, continuous slow-pyrolysis reactors dominate commercial deployment and define the core scaling law derived here. We compiled and harmonized 17 cost observations from 10 studies spanning six continents and adjusted them to a delivered-to-market boundary in constant 2024 USD (Section S1.3; Table S3). After filtering to a core set of standalone slow-pyrolysis systems, an eight-point dataset was retained for empirical cost–capacity scaling.

Across the retained 8-point biochar domain, plant output corresponds approximately to 0.7–89 dry tonnes of biochar per day on a 365-day basis. Using the study-specific char yields reported in Table S1, this corresponds to roughly 2.4–297 dry tonnes of dry feedstock per day. This remains far below the 2,000 dry metric tonnes per day commonly assumed in National Renewable Energy Laboratory (NREL) nth-plant lignocellulosic fuel design cases, reinforcing that the biochar systems studied here are generally more modular and less capital-intensive than commercial-scale fuel biorefineries.^16^

#### Cost-capacity scaling

While declining unit cost with increasing scale is expected in capital-intensive conversion systems, the magnitude and robustness of this effect for biochar production have not been empirically established across harmonized studies. Refitting the eight-point dataset yields the following power-law expression for delivered cost *C* (USD t^−1^ biochar, 2024) as a function of plant capacity *Q* (t biochar yr^−1^):

(Equation 1) $$C_{2024}=1.08\times 10^{4}\;Q^{-0.37}\;\left(\pm 40\%\right),$$

where the exponent represents the elasticity of cost with respect to scale. In practical terms, larger pyrolysis units reduce unit costs mainly by spreading labor, equipment, and overhead over more biochar output; however, this empirical relationship should be interpreted only within the observed scale domain. Within the empirically observed range of 0.1–10 kt yr^−1^, the fitted elasticity implies a nearly 60% decline in delivered cost for each 10-fold increase in capacity. Robustness of the estimated exponent was evaluated using alternative regression approaches (Huber regression), leave-one-out refits, and sensitivity tests to data-filtering thresholds, all of which yield consistent scaling behavior (see Section S1.4 for regression and robustness diagnostics). Uncertainty propagation indicates a delivered-cost confidence band of approximately ±40% that is nearly invariant across scale.

The observed total-cost exponent also aligns in magnitude with classical chemical-engineering scale factors. If installed capital scales approximately with capacity to the 0.6–0.7 power, as in the traditional “six-tenths rule,” then capital cost per unit output scales approximately with capacity to the −0.4 to −0.3 power. Our fitted capital-recovery slope of about −0.30 is consistent with this expectation. The steeper overall delivered-cost slope of −0.37 arises from additional scale effects, particularly labor dilution and, to a lesser extent, utilities, which further reduce per-unit costs with increasing capacity.^17^

#### Drivers of scale effects

Median cost composition across the core set is roughly 40% of total cost to capital recovery, 25% to labor and fixed operating costs, and 30% to fuel and utilities (see Table S4 for detailed cost breakdowns across studies). Component-level regressions clarify the relative drivers of the observed scaling behavior. Labor exhibits the strongest scale sensitivity (cost α *Q*^−0.52^), reflecting quasi-fixed staffing requirements that do not increase linearly with throughput. Capital recovery follows a weaker trend (cost α *Q*^−0.30^), while fuel and utilities decline more modestly with size. Together, these components reinforce the aggregate exponent of −0.37 derived from the total-cost fit. This relationship illustrates classic economies of scale in pyrolysis: as capacity increases, fixed labor and overhead costs are distributed over larger output, and larger reactors achieve higher thermal efficiency and energy recovery.

The scale contrast with SAF is economically important. Pyrolysis units can often be deployed as lower capital expenditure (CAPEX), modular systems whose cost declines mainly through labor dilution, equipment reuse, and better thermal integration. By contrast, FT, ATJ, and CFP SAF facilities couple feed preparation with catalytic upgrading, product separation, and often low-carbon hydrogen supply, creating substantially higher first-of-a-kind capital requirements and greater sensitivity to financing conditions.^18^^,^^19^

#### Economic domains

The scaling law delineates three operational cost regimes relevant to biochar deployment. At capacities below approximately 100 t yr^−1^, micro-scale or seasonal systems fall outside the empirical model and experience prohibitively high unit costs dominated by logistics and manual labor. Between roughly 0.1 and 10 kt yr^−1^ (the “economies-of-scale zone”), internal process efficiencies dominate, leading to rapid cost decline with scale. Beyond 10 kt yr^−1^, transport distance, moisture management, and feedstock aggregation are anticipated to begin offsetting internal gains, creating a “logistics-constrained” regime where total cost rises again.

**Figure 1:**
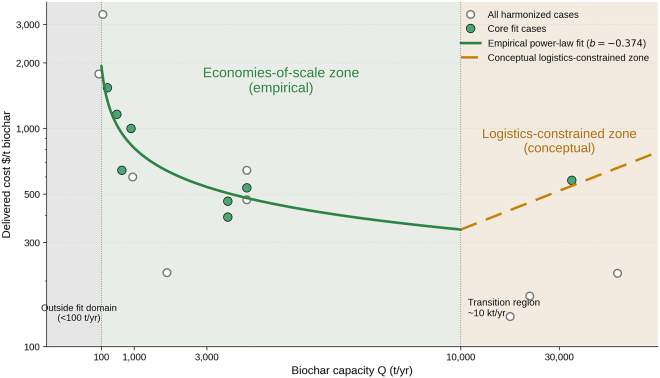
Biochar cost curve showing an empirical economies-of-scale region and a conceptual logistics-constrained region Gray points show all harmonized biochar TEA cases and green points the eight core fit cases. The solid curve shows the empirical power-law fit displayed through 0.1–10 kt yr^−1^; although one retained core observation lies at 32.5 kt yr^−1^, the density of observations above roughly 5 kt yr^−1^ is limited, so larger scale behavior is not treated as a robustly parameterized continuation of the fit. The dashed segment beyond 10 kt yr^−1^ is therefore conceptual and indicates where feedstock aggregation, transport distance, and moisture handling are expected to offset internal economies of scale. The shaded gray region below 100 t yr^−1^ lies outside the empirical fit domain.

This non-empirical upturn is theoretically motivated by the scaling behavior of biomass collection. In an idealized landscape with uniform biomass density and a well-developed transport network, the area accessible for collection increases with the square of the collection radius, while average transport distance increases linearly with radius. Under such conditions, collection cost per unit biomass would be expected to rise sublinearly with scale, approximately with the square root of throughput. Real-world conditions—heterogeneous biomass distribution, imperfect road networks, terrain constraints, and moisture handling—are likely to produce steeper cost increases, bounded between sublinear and linear scaling. As a result, while capital and processing costs continue to decline with scale, feedstock collection costs eventually dominate, yielding a convex total cost curve (Figure 1) and a transition region near 10 kt yr^−1^ where logistical constraints begin to offset internal economies of scale.

Commercial deployment is not limited to stationary reactors. Trailer-mounted and mobile fire-control systems are already used for orchard residues, forest-slash reduction, and wildfire-mitigation projects, although their seasonal utilization and relocation costs keep unit costs well above those of stationary plants.^7^^,^^9^

#### Interpretation and implications

Equation 1 provides a compact analytic engine for estimating delivered biochar costs across realistic plant scales. For instance, at 1 kt yr^−1^ capacity, the model predicts a mean delivered cost of roughly 815 USD t^−1^; at 10 kt yr^−1^, costs fall to about 345 USD t^−1^. These values define the empirical cost envelope relevant to carbon-removal markets operating under typical feedstock and utilization conditions.

The scaling relationship also establishes a bridge between technology design and market deployment. Cost-efficient deployment of biochar for carbon-removal markets is most consistent with operation in the range where internal economies of scale are strongest and logistics remain tractable. The harmonized core dataset includes one retained large-scale case at 32.5 kt yr^−1^, but the density of observations above roughly 5 kt yr^−1^ remains limited. We therefore display the empirical power-law segment only through 10 kt yr^−1^ and treat larger scale behavior conceptually rather than as a robustly parameterized continuation of the fit. Future research on biochar site selection and scale optimization is needed to resolve how regional feedstock distribution, transport constraints, and market access interact to shape feasible deployment scales.

#### Summary

The empirical relationship expressed in Equation 1 captures the dual nature of biochar economics—strong internal economies of scale bounded by real-world logistical constraints. It consolidates heterogeneous TEA findings into a unified cost function (Section S1) with quantified prediction uncertainty (Section S2) that can be integrated directly into global carbon-removal modeling and comparative assessments of biomass-based mitigation pathways. The next section couples this cost scaling with variability in carbon removal to quantify the full spectrum of climate-service values attainable through PyCCS deployment.

### Carbon removal and variability: Why quality matters

The climate-service value of biochar depends not only on its production cost but also on the carbon yield and permanence of storage. Unlike fossil carbon, which remains geologically stable until extracted, the long-term fate of biochar carbon depends on both how much carbon is fixed during pyrolysis and how long that carbon persists once it returns to the soil. Differences in feedstock type and pyrolysis conditions (temperature, heating rate, and residence time) therefore determine the joint outcome of product yield and stability. Quantifying these sources of variability is essential for translating techno-economic performance into durable, verifiable climate benefit.

#### Determinants of yield and permanence

The fraction of feedstock carbon retained as solid biochar varies widely—from roughly 35% for biosolids to about 77% for woody materials under typical slow-pyrolysis conditions.^20^ Processing conditions introduce systematic trade-offs between carbon yield and long-term stability. The hydrogen-to-organic-carbon ratio (H/C_org_) provides a convenient proxy for this trade-off, linking stability to yield losses.^21^ Building on parameter ranges reported in the 2019 Refinement to the Intergovernmental Panel on Climate Change (IPCC) Guidelines for National Greenhouse Gas Inventories,^20^ we used Monte Carlo sampling to represent variability across feedstocks and pyrolysis conditions (see Section S3.1 for the Monte Carlo setup and parameterization). The resulting distributions (Section S3.1; Table S9; Figure S4) indicate that the carbon fraction of biochar expected to remain stable over a century ranges from about 0.23 for biosolids at low temperature (350°C–450°C) to nearly 0.68 for woody feedstocks at high temperature (>600°C). This corresponds to a gross carbon-removal potential of roughly 0.8–2.5 t CO_2_ per tonne of biochar. These spans underscore that biochar is not a single material but a continuum of yields and stabilities defined by feedstock composition and processing conditions.

#### Empirical evidence from audited projects

While IPCC defaults synthesize results from field and laboratory studies to define representative carbon-retention and permanence factors, verified carbon-removal registries provide the clearest view of performance under real operating conditions. Audit data from 19 biochar facilities registered with Puro.earth show net carbon-removal factors derived from issued CO_2_ Removal Certificates (CORCs) between 0.9 and 3.2 t CO_2_ per tonne of biochar, with a mean near 2.3 (Section S3.2; Table S10; Figure S5). These registry values inherently account for both yield and permanence effects and already deduct life cycle emissions from feedstock collection, processing, and transport, thus representing net atmospheric removals. The resulting distribution is slightly right-skewed, reflecting diversity in feedstocks, reactor designs, and operating temperatures. Together, the audited data define the empirical uncertainty envelope for the climate-service performance of PyCCS systems, while the IPCC framework explains the physical and mechanistic basis of the observed variability.

#### Influence on abatement cost

**Figure 2:**
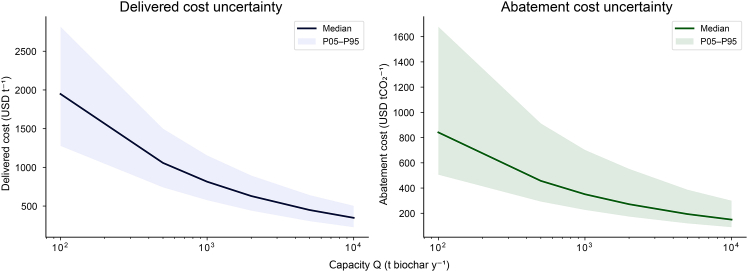
Uncertainty propagation in delivered and abatement cost as a function of production capacity The left image shows delivered cost (USD t^−1^ biochar) including slope and residual uncertainty. The right image converts to abatement cost (USD t^−1^CO_2_) using empirical net CO_2_-removal factors from 19 audited CO_2_ Removal Certificates (CORCs; median ∼2.3 and P_5_–P_95_∼1.6–2.8 t CO_2_t^−1^ biochar; full range 0.9–3.2). Shaded regions denote 5th–95th percentile ranges; solid lines denote median (*P*_50_) estimates. The figure illustrates uncertainty propagation rather than project-specific performance.

Abatement cost is therefore a permanence-weighted metric: delivered cost is divided by net, audited CO_2_ removal rather than by tonnes of biochar alone. A low-cost char with poor permanence can thus yield a worse climate-service cost than a somewhat more expensive but highly stable char. When variability in carbon yield and permanence is combined with statistical uncertainty in the cost-capacity relationship, the resulting abatement-cost distribution is broad yet predictable (Figure 2). Across the 0.1–10 kt yr^−1^ domain, median costs decline from about 840 USD t^−1^CO_2_ at 100 t yr^−1^ to roughly 150 USD t^−1^CO_2_ at 10,000 t yr^−1^. For delivered cost, the 5th–95th-percentile band remains nearly scale-invariant at approximately ±40% around the median. For abatement cost, the band is asymmetric—typically about 40% below and 100% above the median—reflecting the combined effect of cost prediction error and variation in realized CO_2_-removal factors. Deterministic scaling sets the downward trend with capacity, while uncertainty in cost and carbon removal defines the width of the envelope (Figure 2).

#### Implications for climate-service valuation

For carbon-removal markets, combined uncertainty in yield and permanence translates directly into valuation risk. Incorporating these distributions into abatement-cost analysis supports durability-adjusted crediting and risk-informed procurement. From a systems standpoint, this integration shows that apparent variability in biochar economics arises as much from differences in carbon-conversion efficiency and permanence as from production cost. As registry standards mature and analytical verification of both biochar yield and stable-carbon content become routine, uncertainty around removal performance—and thus around climate-service valuation—should narrow substantially. Continued improvement in yield and permanence data will further refine estimates of the climate-service cost of PyCCS and strengthen its integration into broader carbon-removal portfolios.

### Biochar and SAF: The climate-service frontier

Global commercial deployment of biochar has expanded rapidly, with an estimated installed production capacity exceeding 350 kt yr^−1^ across six continental regions as of 2023 (see Section S4.2; Tables S11–S13).^22^ These facilities span from small batch kilns to multi-kilotonne continuous reactors, collectively sampling the full range of the cost-capacity curve derived above.

#### Technology readiness and scaling risk

Commercial readiness further differentiates the near-term climate-service roles of these pathways. Biochar slow-pyrolysis systems are already deployed across a wide range of plant scales and integrated into third-party carbon-removal registries. By contrast, current SAF supply is still dominated by hydroprocessed esters and fatty acids (HEFA) produced from waste oils and fats, while FT, ATJ, and CFP routes derived from lignocellulosic residues or municipal waste remain less mature and more capital intensive. IATA’s 2025 outlook treats HEFA as already commercial but applies success factors and slower rollout assumptions to the other routes because most are not yet at full commercial readiness, and it notes that municipal-solid-waste FT projects have already experienced delays and cancellations.^13^ Recent commercialization reviews and industry reporting likewise document repeated delays, restructuring, or cancellation among first-of-a-kind residue- and waste-based SAF ventures, including widely discussed US projects such as Red Rock and Fulcrum.^18^^,^^19^^,^^23^^,^^24^

This matters for cost comparison. HEFA is constrained mainly by feedstock availability; FT, CFP, and many ATJ configurations face larger first-of-a-kind capital requirements, extensive upgrading, and dependence on low-carbon hydrogen or power, which introduces added cost volatility and infrastructure dependence. Hydrogen supply is a particularly important scaling constraint: hydroprocessing and hydrogenation are integral to several SAF routes, while the PtL/e-SAF systems often invoked for long-run aviation decarbonization depend directly on large-scale electrolytic hydrogen, renewable-power build-out, and water or desalination infrastructure. Recent SAF assessments also note that electrolyzer manufacturing and deployment capacity remains a bottleneck, with supply chain advantages currently concentrated in countries such as PR China. For that reason, our SAF distribution should be read as a cross-technology envelope rather than as a learning curve for any single pathway.^13^^,^^18^^,^^19^

#### Global biochar abatement-cost distribution

Using the empirically fitted scaling law—together with its ±40% model uncertainty—and the observed variability in verified carbon removal from the previous section, we estimate the effective abatement-cost distribution for this global fleet. Median abatement costs range from 164 to 206 USD t^−1^CO_2_ across regions, with 5th–95th-percentile bounds typically between 111 and 497 USD t^−1^. The capacity-weighted global distribution yields a median of 177 USD t^−1^CO_2_ and a 5th–95th range of 111–497 USD t^−1^ (Section S4.6; Table S14). These figures confirm that commercial PyCCS is viable for durable carbon storage within the moderate-cost domain predicted by our scaling model.

#### Comparison with waste-based fuels

**Figure 3:**
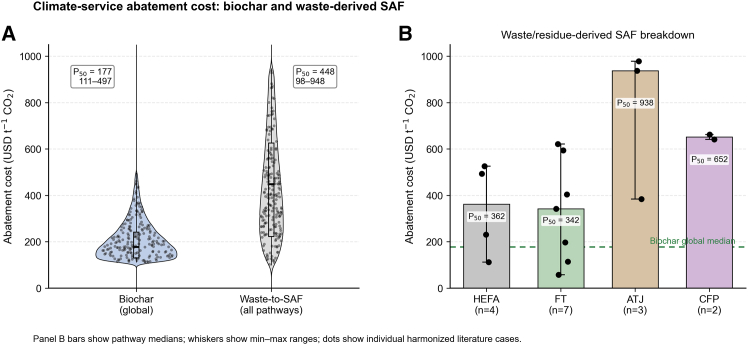
Climate-service cost comparison between biochar and waste-biomass-derived SAF pathways (USD t^−1^CO_2_, 2024) (A) Violin plots show fitted distributions of abatement costs for biochar (global Monte Carlo distribution; median = 177 USD t^−1^CO_2_; P_5_–P_95_ = 111–497 USD t^−1^CO_2_) and for the aggregate waste-to-SAF literature sample (median = 448 USD t^−1^CO_2_; P_5_–P_95_ = 98–948 USD t^−1^CO_2_). (B) Waste/residue-derived SAF pathway breakdown based on 16 harmonized literature cases: bars show pathway medians, whiskers show minimum-maximum ranges, and points show individual cases. The compiled sample comprises HEFA (*n* = 4; median = 362; range = 112–526), FT (*n* = 7; median = 342; range = 57–621), ATJ (*n* = 3; median = 938; range = 384–978), and CFP (*n* = 2; median = 652; range = 641–662). The horizontal dashed line marks the global biochar median. Crop-based starch routes are excluded from the SAF sample to preserve a waste-biomass comparison. All costs are expressed in constant 2024 USD.

Waste-to-SAF pathways occupy a markedly higher-cost regime (Figure 3A). Their aggregate median abatement cost is 448 USD t^−1^CO_2_, with a 5th–95th-percentile span of 98–948 USD t^−1^. SAF abatement costs are computed by combining delivered fuel costs with pathway-specific life cycle carbon intensities relative to a fossil jet baseline (see Section S5), consistent with CORSIA eligible-fuels life cycle accounting conventions.^15^ The compiled waste/residue-derived SAF dataset contains 16 cases: 4 HEFA, 7 FT, 3 ATJ, and 2 CFP. Because ATJ and CFP are represented by only three and two public cases, respectively, pathway medians should be interpreted as descriptive summaries of the compiled cases rather than robust estimates of pathway-level central tendency. The pathway breakdown in Figure 3B shows that HEFA spans 112–526 USD t^−1^CO_2_ (median 362), FT spans 57–621 (median 342), the residue-based ATJ cases reach 384–978 (median 938), and CFP clusters at 641–662 (median 652). This disaggregation shows that the aggregate SAF envelope combines relatively lower-cost but feedstock-constrained HEFA and favorable FT scenarios with substantially higher-cost ATJ and CFP cases that are relevant to larger long-run scale. HEFA’s near-term advantage therefore reflects favorable current feedstock economics rather than long-run scale potential; deep aviation decarbonization ultimately depends more heavily on lignocellulosic and other non-lipid waste routes, which are more challenging to commercialize. Across the compiled SAF studies, the wide spread in abatement cost reflects strong sensitivity to key assumptions, including waste-lipid price, residue logistics, plant-scale design, low-carbon hydrogen cost, and electricity carbon intensity in upgrading steps. Part of the apparent difference between biochar and SAF abatement costs reflects differences in process intensity and carbon efficiency. SAF production generally requires higher temperatures, catalytic upgrading, and hydrogen inputs, increasing both capital and operating costs, while biochar systems retain a larger fraction of biogenic carbon in a stable form. These contrasting mechanisms define the basis of comparison: carbon efficiency relative to the climate service delivered.

Unlike the biochar distribution, which propagates quantified cost and removal uncertainty, the SAF distribution is an empirical envelope across heterogeneous pathways. Its median should therefore be interpreted as descriptive of the current literature sample rather than as a statistically symmetric estimate for any one technology class.

#### Implications for biomass mobilization

The comparison clarifies how biochar and waste-based SAF occupy complementary roles in a broader mitigation strategy rather than competing for a single optimal use of waste biomass. When evaluated on a consistent abatement-cost basis, biochar currently occupies a lower-cost range in this comparison, reflecting its simpler conversion pathway, lower energy requirements, and the large fraction of biogenic carbon that can be stabilized as a durable climate benefit under present technologies. By contrast, waste-derived SAF pathways remain technologically complex and energy-intensive, with production costs strongly shaped by hydrogen availability, catalytic upgrading efficiency, catalyst durability, and stringent fuel-specification requirements. The central insight is therefore not a prescriptive allocation of biomass, but a temporal one: biochar offers a mature, near-term route for mobilizing waste biomass into verifiable removals, while SAF represents a longer term pathway whose climate-service value will grow as technologies mature and costs decline.

## Discussion

### Implications for carbon markets and future research

Our results highlight a central but underexamined point: waste biomass can support multiple mitigation pathways that operate across different time horizons, mitigation capacities, and techno-economic conditions. The dominant institutional mechanism for using waste biomass in aviation—waste-to-SAF under the CORSIA—plays a critical role in decarbonizing a hard-to-abate sector.^14^ However, TEAs consistently show that many SAF routes remain technologically challenging and comparatively costly at present, with economics shaped by feedstock prices, hydrogen requirements, and catalytic upgrading. Viewed on a practical timeline, biochar appears best positioned for scale-up through about 2030, whereas residue-based SAF is more likely to matter materially in the 2030s and 2040s if first-of-a-kind plants achieve reliable operation. This near-term/longer term distinction is conditional on the current evidence base and could narrow if residue- and waste-derived SAF plants demonstrate reliable commercial operation, lower capital intensity, lower low-carbon hydrogen costs, and transparent life cycle performance at scale.

In contrast, biochar exhibits markedly lower abatement costs across the global fleet (Figure 3) and is already technologically mature. PyCCS systems operate today at a wide range of scales, and audited carbon-removal credits demonstrate consistent net removals under real operating conditions. On feasibility grounds, biochar currently offers a near-term option for mobilizing waste biomass into durable climate mitigation while complementary SAF pathways continue to scale and mature. In the broader carbon-removal portfolio, biochar removals can also complement higher cost engineered options such as direct air carbon capture and storage (DACCS)^25^ by expanding near-term supply of durable removals while those technologies continue to mature.

Policy design will strongly shape where marginal waste biomass goes. SAF mandates, tax credits, low-carbon fuel standards, and airline procurement can direct biomass toward aviation fuels even when durable removal appears cheaper on a per-tonne climate-service basis, whereas carbon-removal crediting rewards permanence but does not directly decarbonize fuel demand. Transparent policy therefore needs to recognize the trade-off between near-term, lower cost removals, and longer term fuel-displacement goals rather than assume that all biomass-derived climate services are interchangeable.

Removal credits and SAF life cycle carbon-intensity schemes are also not interchangeable from a certification standpoint. Biochar credits rely on project-level monitoring, reporting, and verification together with permanence accounting, whereas SAF policies rely on pathway life cycle carbon intensity values, book-and-claim systems, and aviation-sector allocation rules. Avoiding double counting is essential when the same feedstock, coproduct, or emissions reduction might otherwise be claimed in both fuel and removal markets.

Counterfactual uses of wastes and residues matter for both pathways. Some biomass streams already displace other products or energy, while others carry tipping fees or coproduct credits that materially alter net cost. Our harmonization preserves the boundary choices reported by source studies rather than impose a universal counterfactual or co-product convention; this keeps the comparison transparent but means the absolute values remain context dependent.

Biochar should also be viewed within a broader biomass carbon removal and storage (BiCRS) portfolio that includes options such as biomass burial or engineered biomass storage. In some contexts those alternatives may offer lower cost or greater permanence, while biochar may provide additional agronomic co-benefits such as soil-water and nutrient retention. SAF can likewise offer co-benefits related to domestic liquid-fuel security and refinery transition. These co-benefits are not monetized here.

### Limitations of the study

This comparison is a current evidence snapshot rather than an intrinsic ranking of biomass pathways. It harmonizes currency year and delivery boundary but retains source-study financing assumptions, co-product conventions, and pathway-specific counterfactuals. The biochar curve is empirical for standalone slow-pyrolysis systems over roughly 0.1–10 kt yr^−1^, whereas the SAF distribution is a 16-point cross-technology envelope. Future public data—especially for lignocellulosic SAF—could narrow or shift both ranges. The most valuable future evidence would be public, pathway-specific operating, and techno-economic data for lignocellulosic and municipal-solid-waste SAF plants, including realized fuel costs, life cycle carbon intensities, hydrogen and electricity inputs, coproduct treatment, financing assumptions, and feedstock-logistics boundaries.

## Resource availability

### Lead contact

Requests for further information and resources should be directed to and will be fulfilled by the lead contact, Hassan El-Houjeiri (hassan.elhoujeiri@kapsarc.org).

### Materials availability

This study did not generate new physical materials.

### Data and code availability

• All data used in this study are tabulated in the main text and supplemental information and will be publicly available as of the date of publication.
• All original code used to reproduce the analyses and figures reported in this paper have been deposited at Zenodo and is publicly available at https://doi.org/10.5281/zenodo.20365487. The repository includes Python scripts, supporting files, and a README file describing how to reproduce the reported calculations and figures.
• Any additional information required to reanalyze the data reported in this paper is available from the lead contact upon request.

## Acknowledgments

This research received no external funding.

## Author contributions

Conceptualization, H.E.-H., A.R.B., and M.S.M.; methodology, H.E.-H. and M.S.M.; formal analysis, investigation, visualization, and writing – original draft, H.E.-H.; writing – review and editing, H.E.-H., A.R.B., and M.S.M.

## Declaration of interests

The authors declare no competing interests.

## STAR★Methods

### Key resources table

**Table:** 

REAGENT or RESOURCE	SOURCE	IDENTIFIER
**Deposited data**		

Harmonized biochar and waste-derived SAF techno-economic dataset (this study)	This paper	Zenodo: https://doi.org/10.5281/zenodo.20365487
Audited biochar net CO2-removal factors from 19 CORC-issuing facilities	Puro.earth registry	https://puro.earth

**Software and algorithms**		

Python 3 (analysis and figure environment)	Python Software Foundation	https://www.python.org
Original analysis and figure-generation code (this study)	This paper	Zenodo: https://doi.org/10.5281/zenodo.20365487

### Experimental model and study participant details

Not applicable. This study synthesizes published techno-economic assessments, public technical reports, and audited carbon-removal registry data and does not involve human participants, animals, cell lines, or newly generated experimental materials.

### Method details

#### Comparison scope and harmonization basis

The analysis compares two different climate services delivered from waste biomass: durable carbon removal via biochar/PyCCS and fossil-jet displacement via waste-derived SAF. All monetary values were harmonized to constant 2024 USD. For biochar, reported minimum selling price or levelized cost of biochar was converted to a delivered-to-market basis by adding 25.5 USD t^−1^ when a study reported gate-only costs. For SAF, abatement cost was computed as the fuel-cost premium relative to fossil jet divided by the pathway-specific life cycle carbon-intensity reduction relative to the fossil baseline. Source-study discount rates, plant lifetimes, and financing conventions were retained rather than re-underwritten across the full literature set.

#### Biochar techno-economic dataset and archetypes

We compiled 20 published biochar TEA scenarios spanning five archetypes: continuous slow pyrolysis, mobile fire-control systems, microwave-assisted pyrolysis, hydrothermal carbonization plus post-carbonization, and CHP-integrated systems. One scenario lacking a reported minimum selling price and two duplicate Monte Carlo summary rows were excluded, leaving 17 harmonized cost observations from 10 studies. For each scenario we recorded reactor type, feedstock, char yield, operating days, original cost year and currency, system boundary, and any reported co-product or gate-fee treatment. Exact facility-level feedstocks, boundaries, and harmonized values are listed in Tables S1–S4.

#### Biochar scaling fit and cost uncertainty

A log–log power law was first fitted to the 17-point harmonized dataset. To isolate representative standalone slow-pyrolysis economics, cases were retained when their absolute residual from the initial fit was ≤30% and coproduct or gate-fee credits were ≤30% of gross cost, leaving eight core cases. The final fit therefore reflects a curated subset intended to capture intrinsic production economics rather than cases dominated by exogenous credits. Parameter uncertainty was quantified using Student-t confidence intervals, robustness was checked using Huber regression and leave-one-out refits, and cost uncertainty was propagated by Monte Carlo over the 0.1–10 kt yr^−1^ domain.

#### Carbon-removal factors and permanence interpretation

Net-removal factors were taken from 19 audited Puro.earth biochar facilities. Each factor represents tonnes of CO_2_ removed per tonne of dry biochar after deduction of upstream emissions under registry methodology. These audited factors were sampled directly with replacement in the main abatement-cost propagation. IPCC default carbon-content and permanence factors were analyzed separately to explain the physical basis of variability in yield and stability, but the main climate-service cost model is parameterized by audited net-removal factors rather than theoretical gross storage.

#### Waste-derived SAF dataset and pathway grouping

We compiled 16 waste- or residue-derived SAF cases spanning HEFA, FT, ATJ, and CFP. The sample includes 4 HEFA, 7 FT, 3 ATJ, and 2 CFP cases. Crop-based starch routes were excluded from the harmonized envelope to preserve a waste-biomass comparison. For each case we extracted the reported fuel cost, life cycle carbon intensity, and reporting year, inflated costs to 2024 USD, and computed abatement cost relative to fossil jet. Because public pathway-specific datasets remain sparse and heterogeneous, the aggregate SAF comparison is treated as a cross-technology envelope rather than as a pathway-specific learning curve.

### Quantification and statistical analysis

Regional and global biochar abatement-cost distributions were generated by combining delivered-cost draws at representative capacities with audited net-removal draws and weighting regional contributions using installed-capacity shares from the 2023 Global Biochar Market Report. The biochar distribution therefore reflects both scale structure and project-level removal heterogeneity.

For SAF, the graphical envelope in Figure 3A was fit to the empirical 5th, 50th, and 95th percentiles of the 16 compiled pathway values. Figure 3B presents the same 16 cases disaggregated by pathway class using pathway medians and minimum–maximum ranges to avoid over-interpreting the aggregate median as a symmetric estimate for any single technology. Across the compiled studies, key drivers of pathway spread include feedstock cost, plant-scale assumptions, low-carbon hydrogen cost, and electricity carbon intensity in upgrading steps.

Source-study discount rates, plant lifetimes, financing assumptions, and co-product conventions were not fully standardized across all TEAs. Instead, these differences remain embedded in the spread of the harmonized literature sample and are made transparent through Table 1, the STAR Methods descriptions, and the SI harmonization tables.

### Additional resources

Further details on facility-level feedstocks, system boundaries, cost breakdowns, filtering thresholds, robustness tests, audited removal factors, and individual SAF cases are provided in Tables S1–S15 and Figures S1–S5 of the supplemental information.

## Supplemental information

Document S1. Figures S1–S5 and Tables S1–S15 and Section S1–S5
