# Supplementary material for: Carbon compensation or fuel displacement? Biomass allocation trade-offs for decarbonizing aviation
Source: iScience. 2026 Jul 16;29(8):116848. doi: 10.1016/j.isci.2026.116848 (PMC13401038; doi:10.1016/j.isci.2026.116848)
Supplement: Document S1. Figures S1–S5 and Tables S1–S15 and Section S1–S5 [file mmc1.pdf]

iScience, Volume *29*

Supplemental information

Carbon compensation or fuel displacement? Biomass allocation trade-offs for decarbonizing aviation

Hassan El-Houjeiri, Adam R. Brandt, and Mohammad S. Masnadi

S1. Dataset harmonization and regression derivation

S1.1 Geographic scope of the techno-economic assessments

A capacity-by-location map (Figure S1) illustrates the geographic clustering of the twenty techno-economic assessments (TEAs) analyzed in this work. The main clusters include: the Pacific Northwest of the United States (forest slash); California's Central Valley (orchard prunings); Central Europe and the United Kingdom (agro-residues, sludge, digestate); Chile (Zimmer 2022[1], sewage sludge); Morocco (Bahiri 2025[2], olive residue); China (swine manure); and Malaysia (palm-oil empty fruit bunch). Marker area is proportional to total annual biochar capacity represented by each regional cluster. If a single study reports multiple scenarios for the same location, the scenario with the highest capacity is used to represent that region on the map.

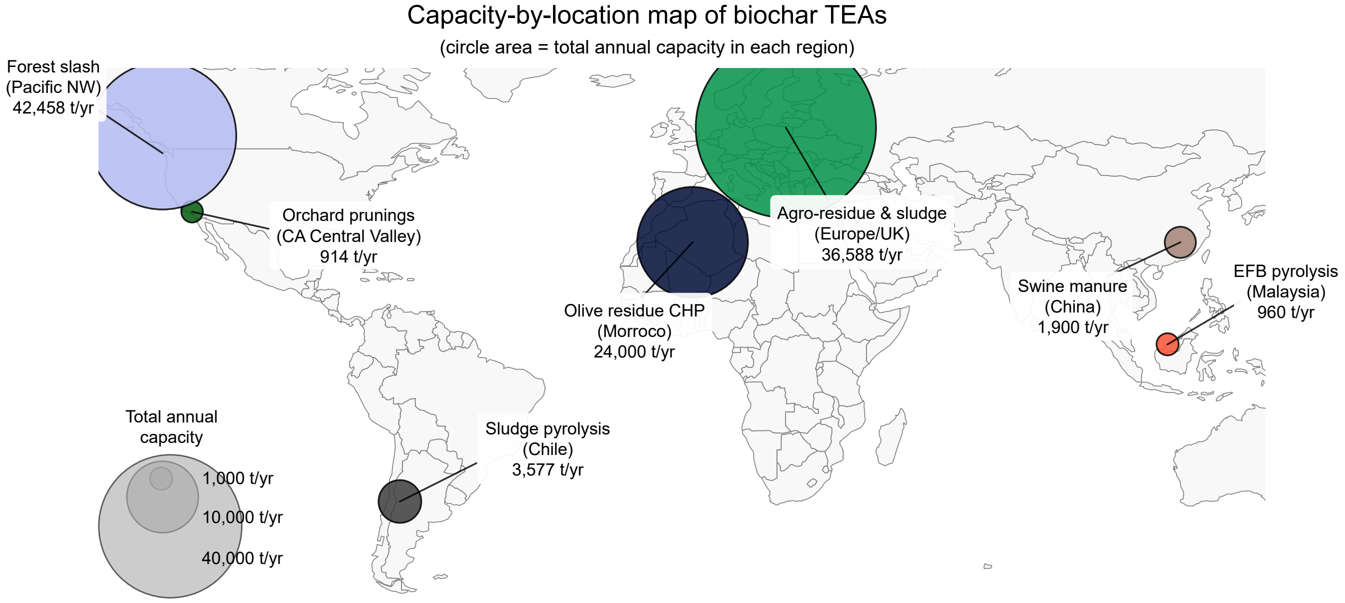

Figure S1: Geographic distribution of the 20 TEAs included in this study. Marker area is proportional to the total annual biochar production capacity represented by each regional cluster.

S1.2 Technology archetypes

The techno-economic assessments (TEAs) compiled in this study encompass five distinct biochar-production archetypes, each characterized by specific process configurations, thermal energy balances, and capital–labor structures. Together these archetypes represent the diversity of commercial and pre-commercial technologies modeled in the 20 TEAs summarized in Table S1.

S1.2.1 Continuous slow pyrolysis (CSP)

Feedstock with a moisture content of 10–20 wt % is metered continuously into a rotating kiln or screw-type reactor. Indirect heating—via recirculated syngas or auxiliary burners—maintains bed temperatures of 450–600 °C under an inert or mildly oxidative atmosphere. Volatile gases are swept out by a carrier gas or under slight vacuum; solid residence times range from minutes to about one hour, producing 25–35 wt % biochar.

Capital cost scales primarily with reactor shell surface area and gas-cleanup systems. Energy self-sufficiency is typically achieved by combusting process syngas ($\approx$ 30 % of the feedstock's higher heating value), and electricity use remains modest ($\approx$ 12–25 kWh t_{feed}^{-1}). Operating labor

is dominated by feed handling and product packaging. Representative TEAs are summarized below.

- **Zimmer 2022** [11]. Models sewage-sludge pyrolysis in Concepción, Chile, comparing two drying regimes—greenhouse solar and natural-gas belt. After adding the \$25.5 t^{-1} delivery adjustment, minimum-selling prices (MSPs) range \$324–\$464 t^{-1} biochar (2024 USD).
- **Haeldermans 2020** [12]. Evaluates conventional slow-pyrolysis (CPS) of tree bark and coffee husks in a 3 t h^{-1} rotary kiln (∼4,100 t y^{-1} biochar). Reported MSPs span \$472–\$535 t^{-1}, depending on feedstock cost (negative for coffee husk vs. positive for bark). Both systems operate as stand-alone CSP plants where syngas combustion provides process heat.

S1.2.2 Mobile fire-Control (MFC) units

Portable biochar systems are designed for forest-slash and wildfire-mitigation projects, enabling partial carbon retention from biomass that would otherwise be openly burned. Operation is constrained by short burning windows and regional air-quality permits. Representative TEAs are summarized below.

- **BSI skid (Sahoo 2020) [5].** Two 1.5 m^3 retorts powered by a diesel genset, producing 136 t y^{-1} biochar. Frequent relocation between timber sales and seasonal utilization (∼100 d y^{-1}) drive high costs: MSP ≈ \$3,004 t^{-1} (2019 USD), inflated to \$3,335 t^{-1} (2024 USD).
- **Oregon flame-cap kiln (Sahoo 2020) [5].** A pyramid-shaped steel kiln where top-fed biomass pyrolysis gases burn above the rim, minimizing smoke. At 95 t y^{-1} capacity, with an eight-person crew operating 120 d y^{-1}, labor dominates cost (≈ \$1,094 t^{-1}), yielding MSP ≈ \$1,604 t^{-1} (2020 USD) or \$1,780 t^{-1} (2024 USD).
- **Air-curtain trench burner (Sahoo 2020) [5].** A refractory-lined pit uses a high-velocity diesel-blower air curtain to trap particulates and reflect heat. Despite low yield (6.5 wt% char), the higher throughput (658 t y^{-1}) and minimal preprocessing produce MSP ≈ \$579 t^{-1} (2019 USD) or \$643 t^{-1} (2024 USD).

S1.2.3 Microwave-assisted slow pyrolysis (MWP)

Haeldermans 2020 [12] modeled a 2.45 GHz microwave reactor processing 11,088 t y^{-1} tree-bark feedstock (≈ 3 t h^{-1}) with a 37% biochar yield (∼4,100 t y^{-1}). The system consumes 11.84 GWh y^{-1} electricity, corresponding to a power cost of 333 t^{-1} biochar at 0.1152 kWh^{-1}. Electricity accounts for $>$ 56% of the MSP (595 t^{-1}; \$644 t^{-1} in 2024 USD). While eliminating external combustion infrastructure, the configuration's electrical intensity dominates its OPEX and limits scalability under commercial tariffs.

S1.2.4 Hydrothermal carbonization + post-carbonization (HTC–PC)

HTC converts wet biomass (digestate, sludge) into hydrochar under subcritical-water conditions (≈ 200 °C, 2–4 MPa), thereby avoiding an energy-intensive drying step. To meet durability criteria for carbon-removal credits, hydrochar is subsequently post-carbonized at 700–800 °C in a rotary kiln.

Gamaralalage 2025 [4] models anaerobic-digestate HTC–PC producing 32.5 wt % biochar yield. A gate-fee credit of –£641 t^{-1} feedstock offsets 88 % of total cost, yielding a net MSP of £88 t^{-1} (\$112 t^{-1} in 2024 USD). Thermal energy demand is moderate, and overall economics are dominated by the tipping-fee structure rather than process efficiency.

S1.2.5 CHP-integrated pyrolysis

Combined-heat-and-power (CHP) systems co-produce electricity and condensable bio-oil alongside biochar, coupling thermal utilization with grid export. Representative TEAs are summarized below.

- **Bahiri 2025 [18].** Integrates a slow-pyrolysis unit with a 2 MWe organic Rankine-cycle (ORC) turbine. Surplus heat is used for drying olive-pit feedstock; condensed bio-oil is sold into refinery markets. Energy and oil coproducts reduce the MSP by $\approx$ 45 % to 134 t^{-1} (\$145 t^{-1} in 2024 USD).
- **Garcia-Perez 2019 [17].** Retrofits a 30 MW grate boiler, reusing existing flue-gas conduits and emission controls. Reported MSP = \$151.5 t^{-1} (2015 USD); inflated to \$217 t^{-1} (2024 USD) after adding \$25.5 t^{-1} for transport and delivery.

S1.2.6 Summary

The five archetypes capture the full cost–performance range of contemporary biochar technologies—from small, labor-intensive mobile units ($\gtrsim$ \$3,000 t^{-1}) to large, CHP-integrated retrofits ($<$ \$250 t^{-1}). Continuous slow-pyrolysis systems dominate commercial practice and define the core subset used to establish the cost–capacity scaling relationship in Section S1.3.

Table S1: Technology and boundary description of the 20 harvested TEAs.

Study (ID)	Q ($\mathrm{t\,y^{-1}}$)	Days $\mathrm{y^{-1}}$	Reactor / heat source	Feedstock	Char yield	System boundary	Notes
Sahoo 20–BSI	136	100	Twin-drum skid, diesel genset	Forest slash	14–16 %	Delivered	100 $\mathrm{d\,y^{-1}}$; seasonal skid[3] Table 5
Sahoo 20–OK	95	120	Flame-cap kiln, natural draft	Forest slash	20.0 %	Delivered	120 $\mathrm{d\,y^{-1}}$; burn window constraint[3] Sec. 2.2.2
Sahoo 20–ACB	658	150	Air-curtain trench, diesel blower	Forest slash	3.5 %	Delivered	150 $\mathrm{d\,y^{-1}}$; inferred from output rate[3] Sec. 2.2.3
Nematian 21 0.5 t/d	130	261	Trailer kiln, propane start	Orchard prunings	30 %	Gate	Mobile; Monte-Carlo mean[4] Sec. 3.1
Nematian 21 1 t/d	261	261	idem	Orchard prunings	30 %	Gate	261 $\mathrm{d\,y^{-1}}$ assumed[4] Sec. 3.1
Nematian 21 2 t/d	522	261	idem	Orchard prunings	30 %	Gate	—
Nematian 21 3.5 t/d	914	261	idem	Orchard prunings	30 %	Gate	—
Wehrenbrecht 24	32 500	330	Slow pyrolysis, syngas-fired	Wheat straw	30–35 %	Delivered	Continuous slow pyrolysis; full logistics chain[5]
Gamaralalage 25	20 000	330	HTC + rotary kiln (750 ℃)	Food-waste digestate	32.5 %	Delivered	Gate fee –641 £ $\mathrm{t^{-1}}$[6]
Zimmer 22 solar	3 577	n/r	Rotary kiln, solar greenhouse	Sewage sludge	57.1 %	Gate	45 € $\mathrm{t^{-1}}$ added[1]
Zimmer 22 belt	3 577	n/r	Rotary kiln, NG belt dryer	Sewage sludge	57.1 %	Gate	45 € $\mathrm{t^{-1}}$ added
Zimmer 22 MC-s	3 577	n/r	idem (MC mean)	Sewage sludge	57.1 %	Gate	Monte Carlo mean, solar drying
Zimmer 22 MC-t	3 577	n/r	idem (MC mean)	Sewage sludge	57.1 %	Gate	Monte Carlo mean, thermal drying
Haeldermans 20 CPS TB	4 100	350	Rotary kiln, syngas-fired	Tree bark	37 %	Delivered	EU grid price; high yield[7]
Haeldermans 20 MWP TB	4 100	350	Microwave, 2.45 GHz	Tree bark	37 %	Delivered	High electricity demand
Haeldermans 20 CPS COF	4 100	350	Rotary kiln, syngas-fired	Coffee husk	28 %	Delivered	Negative feed cost
Garcia-Perez 19 30MW	41 800	310	Power-plant retrofit, grate boiler	Demo wood	15 %	Gate	Power credit[8]
Bahiri 25 CHP	24 000	340	Slow pyrolysis + CHP	Olive-leached residue	29.41 %	Gate	Bio-oil + power credit[2]
Mao Hu 24 swine manure	1 900	333	Pyrolysis (self-heated with syngas)	Swine manure (solid)	23.75 %	Gate	No co-product revenue[9]
Harsono 13 EFB pyrolysis	960	240	Slow pyrolysis (diesel + syngas)	Palm oil empty fruit bunch (EFB)	20 %	Delivered	Syngas reused; no co-product credit[10]

S1.3 Data compilation and cost harmonization

S1.3.1 Data extraction

For each study, we recorded reactor type, heat input mode, feedstock, char yield, operating days, MSP/LCOB, cost breakdowns (if available), and declared system boundaries. When a study reported multiple scenarios, we treated each as a separate observation. These scenarios could differ by production scale, technology configuration (for example, solar versus belt drying in Zimmer 2022, or conventional slow pyrolysis versus microwave pyrolysis in Haeldermans 2020), or by feedstock pricing assumptions. This approach preserves the diversity and statistical nuances of published TEAs rather than reducing them to averaged values, providing a more representative basis for scaling analysis.

Table S2: Raw techno-economic assessment (TEA) cost data as reported in source publications (no harmonization applied).

ID	Study / Scenario	Q ($\mathrm{t\,y^{-1}}$)	Reported MSP/LCOB	Currency / Year
1	Sahoo 20 – BSI skid	136	3 004	USD 2019
2	Sahoo 20 – Oregon kiln	95	1 604	USD 2020
3	Sahoo 20 – Air-Curtain	658	579	USD 2019
4	Nematian 21 – 0.5 t/d trailer	130	—	USD 2021
5	Nematian 21 – 1 t/d trailer	261	1 426	USD 2021
6	Nematian 21 – 2 t/d trailer	522	1 072	USD 2021
7	Nematian 21 – 3.5 t/d trailer	914	920	USD 2021
8	Wehrenbrecht 24 – straw SP	32 500	534.6	EUR 2024
9	Gamaralalage 25 – HTC–PC	20 000	88	GBP 2024
10	Zimmer 22 – solar dry	3 577	339.5	EUR 2024
11	Zimmer 22 – belt dry	3 577	404.5	EUR 2024
12	Zimmer 22 – solar MC mean	3 577	275.5	EUR 2024
13	Zimmer 22 – thermal MC mean	3 577	383.2	EUR 2024
14	Haeldermans 20 – CPS TB	4 100	494.4	EUR 2020
15	Haeldermans 20 – MWP TB	4 100	594.7	EUR 2020
16	Haeldermans 20 – CPS COF	4 100	436.5	EUR 2020
17	Garcia-Perez 19 – 30MW boiler	41 800	151.5	USD 2015
18	Bahiri 25 – olive CHP	24 000	133.97	EUR 2024
19	Mao Hu 24 – swine manure	1 900	193	USD 2024
20	Harsono 13 – EFB pyrolysis	960	545.45	USD 2013

S1.3.2 Cost harmonization

To ensure comparability between studies, the reported costs were adjusted as follows:

- **Inflation indices:** U.S. costs were inflated using CPI-U[11], Eurozone costs with Eurostat[12], and U.K. costs with ONS[13], bringing all values to 2024 price levels.
- **Exchange rates:** Inflated costs were converted using 2024 IRS average rates[14]: 1 EUR = \$1.0822, 1 GBP = \$1.2781.
- **Transport and distribution (T&D):** Where only factory-gate costs were reported, \$25.5 $\mathrm{t^{-1}}$ was added to account for delivery (based on Sahoo 2019[15], updated from 23.00 USD[2019]/t for ∼50 km bulk transport plus field application).

Table S3: Summary of literature scenarios harmonised to delivered-to-market boundary (USD 2024). T&D of \$25.5 t^{-1} added to cases originally reporting factory-gate MSP/LCOB. *Abbreviations:* CPS TB = conventional pyrolysis system (tree bark); MWP TB = microwave-assisted pyrolysis (tree bark); CPS COF = conventional pyrolysis system (coffee husk, negative feed price); EFB = empty fruit bunch.

ID	Study / Scenario	Q ($t\,y^{-1}$)	USD 2024 (delivered)	Technology note
1	Sahoo 20 – BSI skid	136	\$3 335	seasonal mobile, hybrid (diesel/grid average)
2	Sahoo 20 – Oregon kiln	95	\$1 780	flame-cap batch kiln
3	Sahoo 20 – Air-Curtain	658	\$643	forced-air trench burner
4	Nematian 21 – 0.5 t/d trailer	130	—	MSP not reported
5	Nematian 21 – 1 t/d trailer	261	\$1 538	mobile trailer; +\$25.5 T&D
6	Nematian 21 – 2 t/d trailer	522	\$1 162	mobile trailer; +\$25.5 T&D
7	Nematian 21 – 3.5 t/d trailer	914	\$1 001	mobile trailer with credit; +\$25.5 T&D
8	Wehrenbrecht 24 – straw SP	32 500	\$579	slow pyrolysis, wheat straw
9	Gamaralalage 25 – HTC–PC	20 000	\$137.5	HTC + post-carbonisation; +\$25.5 T&D
10	Zimmer 22 – solar dry	3 577	\$393	sludge, solar drying; +\$25.5 T&D
11	Zimmer 22 – belt dry	3 577	\$464	sludge, NG belt dryer; +\$25.5 T&D
12	Zimmer 22 – solar MC mean	3 577	\$324	MC mean, solar; +\$25.5 T&D
13	Zimmer 22 – thermal MC mean	3 577	\$441	MC mean, thermal; +\$25.5 T&D
14	Haeldermans 20 – CPS TB	4 100	\$535	conventional pyrolysis, tree bark
15	Haeldermans 20 – MWP TB	4 100	\$644	microwave pyrolysis
16	Haeldermans 20 – CPS COF	4 100	\$472	negative feed price, coffee husk
17	Garcia-Perez 19 – 30 MW boiler	41 800	\$217	CHP retrofit; +\$25.5 T&D
18	Bahiri 25 – olive CHP	24 000	\$170.5	char + power + bio-oil; +\$25.5 T&D
19	Mao Hu 24 – swine manure	1 900	\$218.5	on-site piggery pyrolysis; +\$25.5 T&D
20	Harsono 13 – EFB pyrolysis	960	\$600	palm EFB, slow pyrolysis

S1.3.3 Cost-breakdown inventory

Table S4 lists all granular cost buckets as reported in the source TEAs. These values are annualised, unadjusted for CPI or FX, and shown as published.

Three consistent trends emerge from these inventories:

Mobile devices. Low annual throughput inflates both labor and CAPEX recovery. The BSI skid allocates \$326 t^{-1} to feed preparation, relocation, repair, and insurance—overshadowing CAPEX recovery. Seasonal operation adds further per-move costs (cranes, trailers, downtime).

Electrified concepts. In Haeldermans' microwave system, electricity accounts for $\sim$56 % of MSP (333 t^{-1} at 0.1152 kWh^{-1}). Procuring power at wholesale parity (0.04 kWh^{-1}) would reduce this to 116 t^{-1}, lowering MSP by 217 t^{-1}, highlighting the potential for grid-responsive, surplus-driven operation.

Sludge and digestate plants. Feedstocks have zero or negative cost, reflecting avoided disposal fees; in the HTC–PC case, the tipping fee credit equals –641 £ t^{-1} (–\$819 2024).

threeparttable

Table S4: Annualised cost breakdowns exactly as published (no CPI or FX adjustment).

ID	Study	Q	Feed+prep	CAPEX	Labour	Fuel+util	T&D	Credit	Other	Total	Curr/yr
1	Sahoo 20 BSI	136	167	1152	1251	184	23.0	0	226	3 004	USD19
2	Sahoo 20 OK	95	7	195	1094	187	23.0	0	98	1 604	USD19
3	Sahoo 20 ACB	658	2	381	90	61	23.0	0	23	579	USD19
4	Nematian 0.5	130	n/r	—	—	—	n/a	0	—	—	USD21
5	Nematian 1.0	261	n/r	755	273	376	n/a	-117 (co-product)	139	1 426	USD21
6	Nematian 2.0	522	n/r	378	273	376	n/a	-94 (co-product)	139	1 072	USD21
7	Nematian 3.5	914	n/r	216	273	376	n/a	-84 (co-product)	139	920	USD21
8	Wehrenbrecht 24	32 500	131.9	108	70	76	28	-78.7 (co-product)	42	534.6	EUR24
9	Gamaralalage 25	20 000	-641	390	n/r	n/r	12	0	308 (fixed OPEX)	88	GBP24
10	Zimmer 22 solar	3 577	0	302	98	101	n/a	-161.7 (bio-oil)	0	339.5	EUR24
11	Zimmer 22 belt	3 577	0	246	98	223	n/a	-161.0 (bio-oil)	0	404.5	EUR24
12	Zimmer 22 MC-s	3 577	n/r	n/r	n/r	n/r	n/r	n/r	n/r	275.5	EUR24
13	Zimmer 22 MC-t	3 577	n/r	n/r	n/r	n/r	n/r	n/r	n/r	383.2	EUR24
14	Haeldermans 20 CPS TB	4 100	n/r	n/r	n/r	n/r	n/r	n/r	n/r	494.4	EUR20
15	Haeldermans 20 MWP TB	4 100	n/r	n/r	n/r	n/r	n/r	n/r	n/r	594.7	EUR20
16	Haeldermans 20 CPS COF	4 100	-131.5	n/r	n/r	n/r	n/r	n/r	n/r	436.5	EUR20
17	Garcia-Perez 19 30MW	41 800	133.4	72.01	47.85	162.68	n/a	-341 (electricity)	76.56	151.5	USD15
18	Bahiri 25 CHP	24 000	26.24	217.76	n/r	n/r	n/a	-110.03 (bio-oil+gas)	0	133.97	EUR24
19	Mao Hu 24 swine manure	1 900	n/r	n/r	n/r	n/r	n/a	0	n/r	193	USD24
20	Harsono 13 EFB pyrolysis	960	n/r	n/r	n/r	n/r	n/r	0	n/r	545.45	USD13

n/r = not reported (component is within the system boundary but not reported separately).
n/a = not applicable (component is not included in the system boundary).

S1.4 Regression results and robustness diagnostics

This section presents the regression model used to establish the cost–capacity relationship for biochar production systems, the filtering and fitting steps applied to the harmonized dataset, and the robustness diagnostics that validate the empirical results.

S1.4.1 Log–Log Power-Law

The relationship between annual biochar production capacity (Q, t y^{-1}) and delivered cost (C, USD t^{-1}, 2024) follows a power law of the form:

$$C = a\,Q^b, \tag{1}$$

where a is the scale coefficient and b is the scaling exponent. Taking the base-10 logarithm linearizes the model:

$$\log_{10} C = b\,\log_{10} Q + \log_{10} a + \varepsilon, \tag{2}$$

Here, ε represents the residual error in log space, assumed to be independent and normally distributed. Ordinary least squares (OLS) regression in this space yields closed-form estimates for both parameters.

S1.4.2 Initial fit on 17 points (STEP 1)

IDs 4, 12, and 13 (missing or duplicate MSPs) were excluded, yielding a 17-point dataset for the initial fit.

Fit results.

$$b_0 = -0.386 \quad (95\%\ \mathrm{CI}\ -0.53,\ -0.25), \qquad R^2_{\log} = 0.699, \qquad \mathrm{MAPE} = 37.0\%.$$

Predicted delivered costs: \$787 t^{-1} at $Q = 1{,}000$ t y^{-1} and \$323 t^{-1} at $Q = 10{,}000$ t y^{-1}.

Table S5: Residuals for the 17-point initial fit (with credit %).

ID	Study / Case	Q_{tpy}	Cost (USD 2024 t^{-1})	$C_{\text{fit,init}}$	Residual	Residual (%)	Credit (%)
8	Wehrenbrecht 2024 – straw SP	32,500.00	579.00	204.90	1.83	182.50	12.80
1	Sahoo 2020 – BSI skid	136.00	3,335.00	1,700.80	0.96	96.10	0.00
19	Mao Hu 2024 – swine manure	1,900.00	218.50	613.90	−0.64	−64.40	0.00
9	Gamaralalage 2025 – HTC–PC	20,000.00	137.50	247.20	−0.44	−44.40	87.90
15	Haeldermans 2020 – MWP TB	4,100.00	644.00	456.10	0.41	41.20	
3	Sahoo 2020 – Air-Curtain	658.00	643.00	924.90	−0.31	−30.50	0.00
18	Bahiri 2025 – olive CHP	24,000.00	170.50	230.40	−0.26	−26.00	45.10
20	Harsono 2013 – EFB pyrolysis	960.00	600.00	799.30	−0.25	−24.90	0.00
7	Nematian 2021 – 3.5 t/d trailer	914.00	1,001.00	814.60	0.23	22.90	8.40
10	Zimmer 2022 – solar dry	3,577.00	393.00	480.80	−0.18	−18.30	32.30
14	Haeldermans 2020 – CPS TB	4,100.00	535.00	456.10	0.17	17.30	
17	Garcia-Perez 2019 – 30 MW boiler	41,800.00	217.00	185.90	0.17	16.70	69.20
5	Nematian 2021 – 1 t/d trailer	261.00	1,538.00	1,322.10	0.16	16.30	7.60
6	Nematian 2021 – 2 t/d trailer	522.00	1,162.00	1,011.40	0.15	14.90	8.10
2	Sahoo 2020 – Oregon kiln	95.00	1,780.00	1,953.70	−0.09	−8.90	0.00
11	Zimmer 2022 – belt dry	3,577.00	464.00	480.80	−0.04	−3.50	28.50
16	Haeldermans 2020 – CPS COF	4,100.00	472.00	456.10	0.04	3.50	23.20

S1.4.3 Filtering thresholds and outlier treatment

Plotting the twenty harmonized minimum selling prices (MSPs) against annual plant capacity on a log–log scale and fitting the model

$$C = a\,Q^b$$

yields preliminary parameters $a = 11.38 \times 10^3$, $b = -0.39$, and $R^2_{\log} = 0.7$. To identify representative cases for regression, the residual for each observation is computed as the relative difference between the observed and modeled values:

$$\text{residual} = \frac{C_{\text{obs}} - C_{\text{fit}}}{C_{\text{fit}}}.$$

A point is considered an outlier when its absolute residual exceeds $\pm 30\,\%$, that is,

$$|\text{residual}| > 0.3.$$

This threshold balances statistical rigor with data scarcity. It is narrow enough to exclude extreme deviations caused by atypical cost structures, yet broad enough to retain a statistically meaningful sample for the regression.

The credit-share filter further excludes cases in which coproduct or gate-fee credits exceed 30 % of the gross cost, ensuring that retained data primarily reflect intrinsic production economics rather than exogenous subsidies. Applying both filters ($|\text{residual}| \leq 0.30$ and credit $\leq$ 30 %) yields the eight-point core dataset used for the final regression analysis (Table S3).

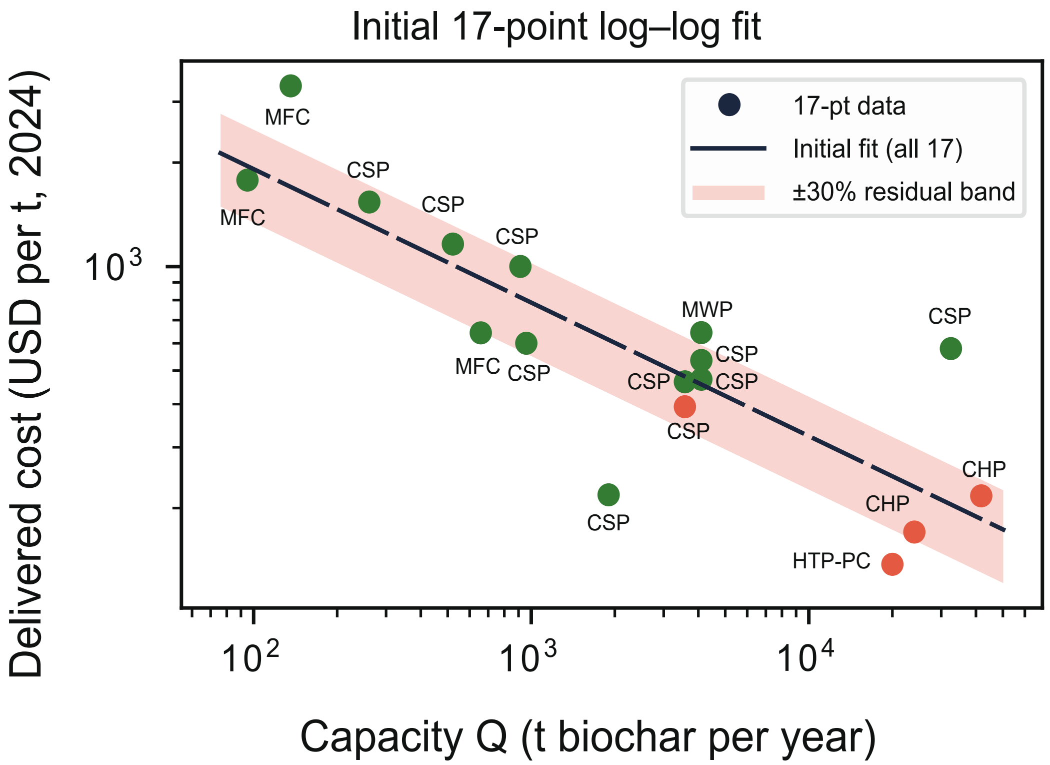

Figure S2: **Initial log–log fit.** Initial 17-point dataset showing the delivered cost ($/t biochar, 2024 USD) versus annual production capacity (Q, t biochar y^{-1}) across multiple biochar technology archetypes. Each point is labeled by archetype—mobile fire-control (MFC), continuous slow-pyrolysis (CSP), microwave-assisted pyrolysis (MWP), hydrothermal carbonization with post-carbonization (HTC–PC), and CHP-integrated systems. Points shaded in green meet the $\leq$30% coproduct-credit threshold used for inclusion, while those in red exceed it and were excluded from the core analysis. The dashed line represents the initial 17-point log–log fit with a $\pm$30% residual band.

S1.4.4 Core dataset refit (STEP 2)

Applying the filters $|\text{residual}| \leq 0.30$ and credit $\leq 30\,\%$ yields an eight-point core dataset (Table S3). The refit gives:

$$b_{\text{core}} = -0.374 \quad (95\% \text{ CI } -0.49,\ -0.25), \qquad R^2_{\text{log}} = 0.91, \qquad \text{MAPE} = 13.5\%.$$

Predicted delivered costs:

$$C_{1\,\text{kt}} = \$815/t\ (95\% \text{ CI}: -117/+136), \qquad C_{10\,\text{kt}} = \$344/t\ (95\% \text{ CI}: -95/+130).$$

Table S6: Core dataset used for final regression (eight observations).

ID	Study / Scenario	Q_{tpy}	Cost (USD 2024 t^{-1})
3	Sahoo 2020 – Air-Curtain	658.00	643.00
5	Nematian 2021 – 1 t/d trailer	261.00	1,538.00
6	Nematian 2021 – 2 t/d trailer	522.00	1,162.00
7	Nematian 2021 – 3.5 t/d trailer	914.00	1,001.00
8	Wehrenbrecht 2024 – straw SP	32,500.00	579.00
10	Zimmer 2022 – solar dry	3,577.00	393.00
11	Zimmer 2022 – belt dry	3,577.00	464.00
14	Haeldermans 2020 – CPS TB	4,100.00	535.00

S1.4.5 Parameter uncertainty of the scaling exponent

The **parameter uncertainty** reported here refers exclusively to the scaling exponent b in the power-law cost–capacity model (Eq. 1). This quantity determines the sensitivity of delivered cost to production scale and therefore dominates the behavior of the regression model. Uncertainty in the intercept a is not discussed, as its effect on cost predictions is a uniform multiplicative scaling and less influential on model sensitivity.

Because the core dataset contains only $n = 8$ observations, the sampling variability of the fitted slope b must be evaluated using the **Student-t** distribution, which accounts for additional uncertainty arising from limited degrees of freedom. For small samples, the t distribution provides wider and more conservative confidence bounds than the normal (z) distribution, thereby avoiding underestimation of parameter uncertainty.

The standard error of the slope is calculated as

$$\mathrm{SE}_b = \frac{s_\varepsilon}{\sqrt{S_{xx}}}, \qquad s_\varepsilon = \sqrt{\frac{\sum_i (y_i - \hat{y}_i)^2}{df}},$$

where s_ε is the residual standard error, $S_{xx} = \sum_i (x_i - \bar{x})^2$ is the total variance in $\log_{10} Q$, and $df = n - 2$ reflects the two fitted parameters (a and b). The $95\,\%$ confidence interval for the slope parameter is then

$$b_{\text{lo,hi}} = b \pm t_{0.975,df}\, \mathrm{SE}_b,$$

where $t_{0.975,df}$ is the two-tailed critical value of the t distribution enclosing $95\,\%$ of its probability mass. For $df = 6$, $t_{0.975,6} = 2.447$.

Applying these relationships to the core dataset gives

$$b = -0.374, \qquad \mathrm{SE}_b = 0.049, \qquad 95\,\%\ \mathrm{CI} = [-0.49,\ -0.25].$$

This confidence interval defines the range within which the *true population value* of the scaling exponent b is expected to lie with $95\,\%$ confidence. It characterizes only the precision of the slope estimate itself and does *not* include the additional uncertainty associated with model residual variance or the prediction of specific cost values. The latter (model-prediction uncertainty) is treated separately.

S1.4.6 Robustness diagnostics and alternative fits

Two robustness methods were used:

- **Huber regression.** A robust estimator that minimizes a hybrid loss function, quadratic for small residuals and linear for outliers, reducing sensitivity to leverage points. Huber regression yielded $b_{\mathrm{Huber}} = -0.374$, identical within rounding to the OLS slope.
- **Leave-One-Out (LOO) analysis.** Repeated refits are performed omitting one observation at a time. The maximum deviation of slopes across iterations was $\max|\Delta b| = 0.034$, with standard deviation $\sigma_b = 0.017$, confirming stability of the fit.

S1.4.7 Summary of sensitivity tests

Credit-threshold and residual-band filters were varied to evaluate the sensitivity of b_{core}. Results are summarized in Table S7.

Table S7: Sensitivity of the scaling exponent to filter thresholds.

Test	Slope b (95% CI)
Core fit (baseline)	–0.374 [–0.49, –0.25]
20 % credit filter	–0.352 [–0.56, –0.15]
40 % credit filter	–0.394 [–0.51, –0.28]
Residual ±25 %	–0.374 [–0.49, –0.25]
Residual ±35 %	–0.365 [–0.50, –0.23]

Interpretation. The scaling exponent ($b = -0.374$) implies a ∼60 % cost reduction per ten-fold increase in capacity. Huber and LOO analyses confirm that no single observation exerts undue influence, and the exponent remains consistent under all filter thresholds. The fitted 95 % confidence envelope is approximately scale-invariant, corresponding to relative deviations of –14 % / +17 % at 1 kt y^{-1} and –28 % / +38 % at 10 kt y^{-1}. Taking the median of these fractional bounds yields a representative scale-invariant uncertainty of approximately $\pm 24\,\%$ across the 0.1–10 kt y^{-1} range.

S2 Cost uncertainty and Monte Carlo propagation

To quantify how uncertainty in model parameters affects predicted biochar costs, we performed a Monte Carlo (MC) simulation based on the fitted cost–capacity relationship

$$C = a\,Q^b,$$

where a and b are the intercept and slope obtained from the core regression (Section S1.4). Two independent sources of uncertainty were included: (i) statistical uncertainty in the fitted slope b, which controls how cost changes with scale, and (ii) residual variance s_ε, which represents unmodeled variability across published techno-economic assessments (TEAs). These effects were combined to obtain a realistic confidence range for delivered cost as a function of plant capacity.

S2.1 Monte Carlo design

Each Monte Carlo iteration followed the steps summarized below. All random draws were produced using a fixed seed (`rng = 2031`) to ensure exact reproducibility.

1. **Randomly vary the slope b.** The slope b was drawn from a *Student-t* distribution with six degrees of freedom. This distribution is commonly used for small sample sizes because it gives wider (more conservative) uncertainty bounds than a normal distribution. We limited the draws to the central 95% range, corresponding to $t_{0.975,6} = 2.447$; in other words, we ignored only the most extreme 5% of outliers.

   $$b^{(k)} = b + z_b^{(k)}\,\mathrm{SE}_b, \qquad z_b^{(k)} \sim t(df{=}6,\ |t| \le 2.45),$$

   where SE_b is the standard error of the slope. These draws represent the statistical uncertainty in the cost–capacity scaling exponent.

2. **Re-anchor the intercept to the data centroid.** To prevent artificial bias between slope and intercept, each slope draw was "pivot-anchored" so that all simulated cost curves intersect the fitted regression line at the mean of the data:

   $$a_{\log}^{(k)} = a_{\log} + b\,x_* - b^{(k)}x_*, \qquad x_* = \overline{\log_{10} Q}.$$

   This step ensures that random changes in slope do not shift the entire cost curve up or down on average.

3. **Add random model scatter.** For each plant capacity Q, we added an independent random term drawn from the same truncated t distribution, scaled by the residual standard deviation s_ε:

   $$r^{(k)} \sim t(df{=}6,\ |t| \le 2.45)\, s_\varepsilon.$$

   This term mimics the real-world variability in reported TEA results that is not captured by the regression itself.

4. **Compute total simulated costs.** Combining the random slope and residual gives the simulated delivered cost:

   $$\log_{10} C^{(k)}(Q) = a_{\log}^{(k)} + b^{(k)} \log_{10} Q + r^{(k)}, \qquad C^{(k)}(Q) = 10^{\log_{10} C^{(k)}(Q)}.$$

   Repeating this process produces an ensemble of cost predictions $C^{(k)}(Q)$ for each capacity value.

A total of $N = 2 \times 10^5$ realizations were generated for each of six representative capacities (100, 500, 1 000, 2 000, 5 000, and 10 000 t y^{-1}).

S2.2 Combining parameter and model variance

Separate simulations were first run for slope uncertainty and for residual uncertainty to examine their individual effects. In the final analysis, both components were combined additively in cost space after removing any median offset:

$$C_{\mathsf{sum}}(Q) = C_0(Q) + [C_{\mathsf{slope}}(Q) - \tilde{C}_{\mathsf{slope}}(Q)] + [C_{\mathsf{resid}}(Q) - \tilde{C}_{\mathsf{resid}}(Q)],$$

where tildes denote median values across all MC realizations and $C_0(Q)$ is the deterministic baseline prediction. Subtracting each component's median keeps the ensemble centered on the best-fit regression while combining the spreads from both uncertainty sources.

S2.3 Delivered-cost uncertainty results

Table S8 summarizes the 5th, 50th, and 95th percentiles of the delivered-cost distributions (USD t^{-1}) obtained from the combined Monte Carlo simulation (200,000 draws).

Table S8: Monte Carlo results for delivered-cost uncertainty (sum method, 200,000 draws).

Capacity Q (t y^{-1})	P05	P50 (median)	P95
100.00	1,277.30	1,947.50	2,815.80
500.00	742.00	1,057.40	1,498.80
1,000.00	575.90	814.70	1,153.70
2,000.00	441.20	629.50	893.90
5,000.00	305.30	449.10	641.40
10,000.00	227.20	348.00	503.10

The Monte Carlo analysis shows that both parameter (slope) and residual (model) uncertainties contribute to total cost variability. At a small-scale capacity of 100 t y^{-1}, the 5th and 95th percentiles deviate from the median by –34.4 % and +44.6 %, respectively; at 10 kt y^{-1}, the deviations are –34.7 % and +44.6 %. Averaging these fractional bounds across the two extremes gives mean deviations of –34.6 % and +44.6 %; taking the midpoint yields an approximately symmetric uncertainty envelope of $\pm 40\,\%$ around the median prediction.

Decomposition of the two components clarifies their relative magnitude. The slope parameter alone produces a 95 % confidence band of approximately $\pm 24\,\%$ around the median delivered cost. The residual term—representing unmodeled variability among TEAs—yields a wider band of approximately $\pm 36\,\%$ when averaged between the smallest (100 t y^{-1}) and largest (10 kt y^{-1}) capacities. When both effects are combined additively in cost space, the total envelope expands modestly to $\pm 40\,\%$. Thus, residual variance accounts for most of the overall spread, with slope uncertainty contributing a smaller incremental broadening of the prediction interval.

Figure S3 illustrates the resulting $\pm 40\,\%$ prediction band across the empirical capacity domain (0.1–10 kt y^{-1}). The shaded region represents the 90 % prediction interval (P05–P95) around the median regression line, which remains nearly scale-invariant across plant scales.

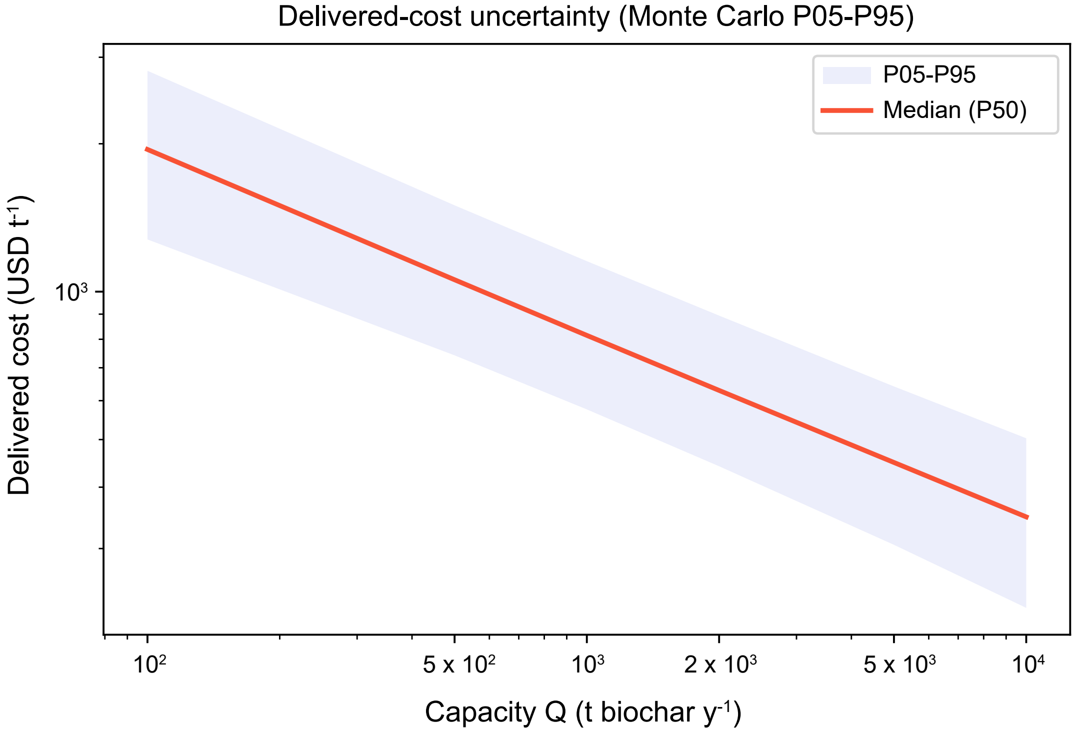

Figure S3: **Delivered-cost uncertainty envelope from the Monte Carlo simulation.** Shaded region shows the 90 % prediction interval (P05–P95) around the median (solid line) on a log–log scale. The uncertainty band is nearly symmetric and scale-invariant, corresponding to an overall $\pm 40\,\%$ confidence range across 0.1–10 kt y^{-1}.

S3 Variability in carbon removal efficiency and empirical CORC distribution

S3.1 IPCC-based variability in biochar carbon content and permanence

To analyze intrinsic variability in the gross carbon-storage potential of biochar across feedstocks and pyrolysis temperatures, we implemented a Monte Carlo simulation based on the default parameters reported in the *2019 Refinement to the 2006 IPCC Guidelines for National Greenhouse Gas Inventories, Volume 4, Appendix 4 ("Biochar")*. The analysis uses values from Table 4AP.1 for the organic-carbon fraction in biochar (F_{Cp}) by feedstock and from Table 4AP.2 for the 100-year permanence fraction (F_{perm}) by temperature band[16].

The IPCC's reported $\pm 95\,\%$ confidence intervals were interpreted as bounds on the mean. For a reported mean μ with relative width r, the standard error was calculated as $\mathrm{SE} = r\,\mu/1.96$. Each parameter was then sampled $N = 100{,}000$ times from a truncated normal distribution using a fixed random seed to ensure reproducibility. Physical truncation limits were applied as follows: $F_{\mathrm{Cp}} \in [0, 1.2]$ (to avoid artificial tail-clipping for wide uncertainty ranges) and $F_{\mathrm{perm}} \in [0, 1.0]$ (strict physical bound). Stable-carbon fraction and gross CO_2-removal potential were computed as

$$F_{\mathrm{stable}} = F_{\mathrm{Cp}} \times F_{\mathrm{perm}}, \qquad \mathrm{Gross\ CO_2/t_{biochar}} = F_{\mathrm{stable}} \times \frac{44}{12}.$$

All realized F_{stable} values remained ≤ 1 in the simulation ensemble.

Table S9 summarizes the resulting means, standard deviations, and percentile bounds for each feedstock and temperature class. Across the six feedstocks, F_{Cp} spans approximately 0.35

(biosolids) to 0.77 (wood), consistent with IPCC defaults. Combining these with the temperature-dependent permanence factors yields stable-carbon fractions of roughly 0.23–0.68 and corresponding gross CO_2 storage potentials of 0.84–2.51 t CO_2 t^{-1} biochar (P05–P95).

Table S9: Monte Carlo results for IPCC-based variability in biochar carbon content, stability, and gross CO_2 removal (100,000 samples per combination; F_{Cp} clipped at 1.2 and F_{perm} clipped at 1.0).

Feedstock	Quantity	Temperature band	Mean	Std	P05	P50	P95
Wood	Biochar C (F_{Cp})	—	0.77	0.17	0.50	0.77	1.04
	Stable C ($F_{\mathrm{Cp}} \times F_{\mathrm{perm}}$)	High ($> 600°$C)	0.68	0.15	0.44	0.68	0.94
	Gross t CO_2/t biochar	High ($> 600°$C)	2.51	0.56	1.60	2.50	3.45
	Stable C ($F_{\mathrm{Cp}} \times F_{\mathrm{perm}}$)	Medium (450–600 °C)	0.62	0.14	0.39	0.62	0.84
	Gross t CO_2/t biochar	Medium (450–600 °C)	2.26	0.50	1.44	2.25	3.10
	Stable C ($F_{\mathrm{Cp}} \times F_{\mathrm{perm}}$)	Low (350–450 °C)	0.50	0.11	0.32	0.50	0.69
	Gross t CO_2/t biochar	Low (350–450 °C)	1.84	0.42	1.16	1.83	2.54
Nut shells/pits	Biochar C	—	0.74	0.15	0.50	0.74	0.98
	Stable C	High ($> 600°$C)	0.66	0.14	0.44	0.66	0.89
	Gross t CO_2/t biochar	High ($> 600°$C)	2.41	0.51	1.60	2.40	3.26
	Stable C	Medium (450–600 °C)	0.59	0.12	0.39	0.59	0.80
	Gross t CO_2/t biochar	Medium (450–600 °C)	2.17	0.45	1.44	2.17	2.92
	Stable C	Low (350–450 °C)	0.48	0.10	0.32	0.48	0.65
	Gross t CO_2/t biochar	Low (350–450 °C)	1.76	0.38	1.16	1.76	2.40
Herbaceous res.	Biochar C	—	0.65	0.15	0.41	0.65	0.90
	Stable C	High ($> 600°$C)	0.58	0.14	0.36	0.58	0.81
	Gross t CO_2/t biochar	High ($> 600°$C)	2.12	0.51	1.31	2.11	2.96
	Stable C	Medium (450–600 °C)	0.52	0.12	0.32	0.52	0.72
	Gross t CO_2/t biochar	Medium (450–600 °C)	1.91	0.45	1.18	1.90	2.66
	Stable C	Low (350–450 °C)	0.42	0.10	0.26	0.42	0.60
	Gross t CO_2/t biochar	Low (350–450 °C)	1.55	0.38	0.95	1.54	2.18
Rice husks/straw	Biochar C	—	0.49	0.10	0.32	0.49	0.66
	Stable C	High $> 600°$C)	0.44	0.10	0.28	0.43	0.60
	Gross t CO_2/t biochar	High ($> 600°$C)	1.60	0.35	1.03	1.59	2.19
	Stable C	Medium (450–600 °C)	0.39	0.09	0.26	0.39	0.53
	Gross t CO_2/t biochar	Medium (450–600 °C)	1.44	0.31	0.93	1.43	1.96
	Stable C	Low (350–450 °C)	0.32	0.07	0.20	0.32	0.44
	Gross t CO_2/t biochar	Low (350–450 °C)	1.17	0.26	0.75	1.16	1.61
Animal manure	Biochar C	—	0.38	0.10	0.22	0.38	0.54
	Stable C	High ($> 600°$C)	0.34	0.09	0.20	0.34	0.48
	Gross t CO_2/t biochar	High ($> 600°$C)	1.24	0.32	0.72	1.23	1.77
	Stable C	Medium (450–600 °C)	0.30	0.08	0.18	0.30	0.43
	Gross t CO_2/t biochar	Medium (450–600 °C)	1.12	0.29	0.65	1.11	1.59
	Stable C	Low (350–450 °C)	0.25	0.07	0.14	0.25	0.36
	Gross t CO_2/t biochar	Low (350–450 °C)	0.91	0.24	0.53	0.90	1.30
Biosolids	Biochar C	—	0.35	0.07	0.23	0.35	0.47
	Stable C	High ($> 600°$C)	0.31	0.07	0.20	0.31	0.42
	Gross t CO_2/t biochar	High ($> 600°$C)	1.14	0.25	0.75	1.14	1.55
	Stable C	Medium (450–600 °C)	0.28	0.06	0.18	0.28	0.38
	Gross t CO_2/t biochar	Medium (450–600 °C)	1.03	0.22	0.68	1.03	1.39
	Stable C	Low (350–450 °C)	0.23	0.05	0.15	0.23	0.31
	Gross t CO_2/t biochar	Low (350–450 °C)	0.84	0.18	0.54	0.83	1.14

Across all feedstocks, the high-temperature band ($> 600°$C) produces the highest mean permanence ($F_{\mathrm{perm}} \approx 0.89$) and stable-carbon fractions ($\bar{F}_{\mathrm{stable}} \approx 0.52 \pm 0.14$), corresponding to an average gross removal of $\sim$1.9 ± 0.6 t CO_2 t^{-1} biochar. Medium-temperature systems (450–600 °C) achieve $F_{\mathrm{stable}} \approx 0.46 \pm 0.12$ and 1.7 ± 0.5 t CO_2 t^{-1}, while low-temperature systems (350–450 °C) decline to $F_{\mathrm{stable}} \approx 0.35 \pm 0.10$ and 1.3 ± 0.4 t CO_2 t^{-1}. These patterns are illustrated in Figure S5.

Feedstock composition strongly influences F_{Cp}, with high-lignin woody and nut-shell materials producing carbon-dense, aromatic chars and ash- or nutrient-rich feedstocks (e.g., biosolids and manure) producing more labile material with lower fixed-carbon yield. Temperature primarily controls F_{perm} through the degree of aromatic condensation: higher peak pyrolysis temperatures yield more persistent carbon fractions but reduce total char yield. The resulting ensemble (Ta-

ble S9, Figure S4) shows stable-carbon fractions spanning ∼0.23–0.68 and corresponding gross CO_2 removal potentials of ∼0.8–2.5 t CO_2 t^{-1} biochar (P05–P95). These distributions represent the realistic variability in carbon-retention efficiency across feedstocks and temperature bands. They are used to illustrate the uncertainty envelope of biochar carbon stability, whereas the subsequent climate-service cost model (Section S4) is parameterized independently from verified CORC data rather than the IPCC-based simulation.

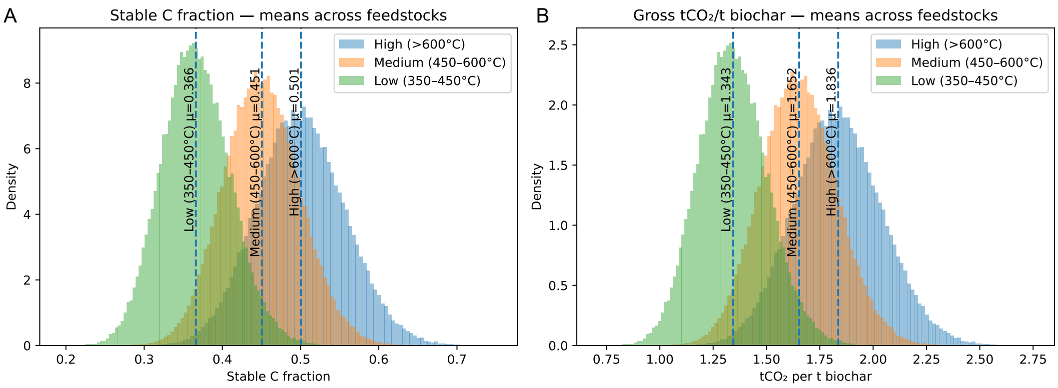

Figure S4: Monte Carlo distributions of (**A**) mean stable-carbon fraction (F_{stable}) and (**B**) mean gross CO_2 removal (t CO_2 t^{-1} biochar) across feedstocks for each IPCC temperature band. Shaded areas denote 5th–95th percentile envelopes of 100 000 draws; dashed lines mark mean values.

S3.2 Audited net removal (CORC) distribution

To capture *realized* net CO_2 removals from operational biochar systems, we assembled audited carbon-removal factors from the *Puro.earth* registry for 19 distinct facilities across Europe, North America, South America, Africa, and Oceania. Each CORC factor is an independently audited net CO_2 removal intensity in t CO_2 per tonne of *dry* biochar, with upstream emissions (collection, preprocessing, transport, application) already deducted under the Puro methodology. Where multiple audit sub-periods were available for the same facility, we calculated the average factor (e.g., Novocarbo Dörth, Germany). In one case (ReGenerate, Oregon, USA) the CORC factor was calculated directly from the audit statement by dividing the number of CORCs issued by the audited biochar tonnage. Only the most recent audited period per facility was retained to avoid duplication. In total, 19 distinct facilities were retained, spanning approximately 0.90–3.18 t CO_2 t^{-1} biochar, with an empirical mean of ∼2.30 and a 5th–95th percentile range of ∼1.60–2.80 t CO_2 t^{-1}. This variability reflects differences in reactor design, operating temperature, feedstock mix, and site context (see Table S10). Audited CORC factors represent net atmospheric CO_2 removals after deduction of life-cycle emissions, and are therefore not directly comparable to the gross CO_2 storage potentials derived from IPCC-based carbon-content and permanence factors in Section S3.1.

Table S10: Audited net CO_2 removal factors (CORC) used in the empirical CORC distribution. Citations refer to auditor reference list.

Project / Facility (country)	Most recent audit period	Auditor (VVB)	Feedstock / notes	CORC	Source / Notes
Carbon Cycle GmbH & Co KG – Rieden (DE)	01 Oct 2023–31 Aug 2024	bio.inspecta AG	Untreated wood chips from local forestry	2.916	Puro audit [17]
Novocarbo – Baltic Sea (DE)	11 Feb 2024–30 Jun 2024	bio.inspecta AG	Wood chips (declared waste; LPG startup noted)	2.37	Puro audit [18]
Novocarbo – Dörth/Rhine (DE)	28 Sep 2022–10 Jun 2023	bio.inspecta AG	—	**2.405**	Average of four audited subperiods (2.310, 2.407, 2.386, 2.517) [19]
Freres Engineered Wood – Lyons, OR (US)	01 Jul 2022–31 Aug 2023	EnergyLink Services	—	2.4736	Puro audit [20]
Exomad SRL – Exomad Green, Concepción (BO)	22 Mar 2024–24 Mar 2025	EnergyLink Services	—	2.517	Puro audit (latest statement) [21]
Planboo ECO AB – Farm Gai Kaisa 159 (NA)	21 Feb 2024–24 Jan 2025	Earthood	Encroacher bush; PV added	2.06	Puro audit [22]
Bio-Logical Mt Kenya – Macadamia (KE)	22 Mar 2024–28 Feb 2025	EnergyLink Services	Macadamia	2.231	Puro audit (eligible factor) [23]
Bio-Logical Mt Kenya – Prosopis (KE)	22 Mar 2024–28 Feb 2025	EnergyLink Services	Prosopis	1.841	Puro audit (eligible factor) [23]
Oregon Biochar Solutions – White City, OR (US)	01 Jul 2022–30 Sep 2022	EnergyLink Services	“Primary Biochar” stream	1.80	Puro audit [24]
Glanris – Olive Branch, MS (US)	01 Apr 2021–31 Aug 2022	EnergyLink Services	—	0.896	Puro audit [25]
Aperam Bioenergia (BR)	01 May 2023–29 Feb 2024	EnergyLink Services	Eucalyptus residues; on-site application	1.654	Puro audit [26]
Pyrocal Pty Ltd – Wellcamp (AU)	01 Jan 2022–31 Jul 2023	EnergyLink Services	— (client-dependent E_{use})	1.895	Puro audit [27]
Nordgau Carbon GmbH & Co. KG (DE)	01 Apr 2022–31 Mar 2023	bio.inspecta AG	—	2.759	Puro audit [28]
Bussme Biochar AB – Munka-Ljungby (SE)	01 Jan 2023–31 Jan 2024	DNV	—	2.547	Puro audit [29]
Bussme Biochar AB – Svedala (SE)	01 Jan 2023–29 Feb 2024	DNV	—	2.789	Puro audit [30]
Carbofex Oy – Nokia 1 (FI)	01 Sep 2022–28 Feb 2023	DNV	—	3.184	Puro audit [31]
Groupe Bordet – Leuglay (FR)	22 Sep 2022–30 Apr 2023	bio.inspecta AG	—	2.659	Puro audit [32]
Carbonchip Mobile Carboniser (AU)	12 Oct 2021–20 Dec 2023	RSM Australia	Mobile carboniser	2.67	Puro audit [33]
ReGenerate (Restoration Fuels – John Day, OR, US)	01 Nov 2023–31 Mar 2024	350Solutions	Forest & mill waste	**2.02**	Implied from 877 CORCs over 434.8 t biochar [34]

No statistical fitting, truncation, or biasing is applied to these data. In all Monte-Carlo analyses, values are drawn directly from the empirical CORC array with replacement. This approach preserves the observed heterogeneity among audited projects and avoids parametric assumptions that are not used elsewhere in the abatement-cost model.

Figure S5 shows the empirical histogram of the 19 audited CORC factors ($t\,CO_2\,t^{-1}$ biochar). The dashed vertical lines mark the empirical 5th, median, and 95th percentiles.

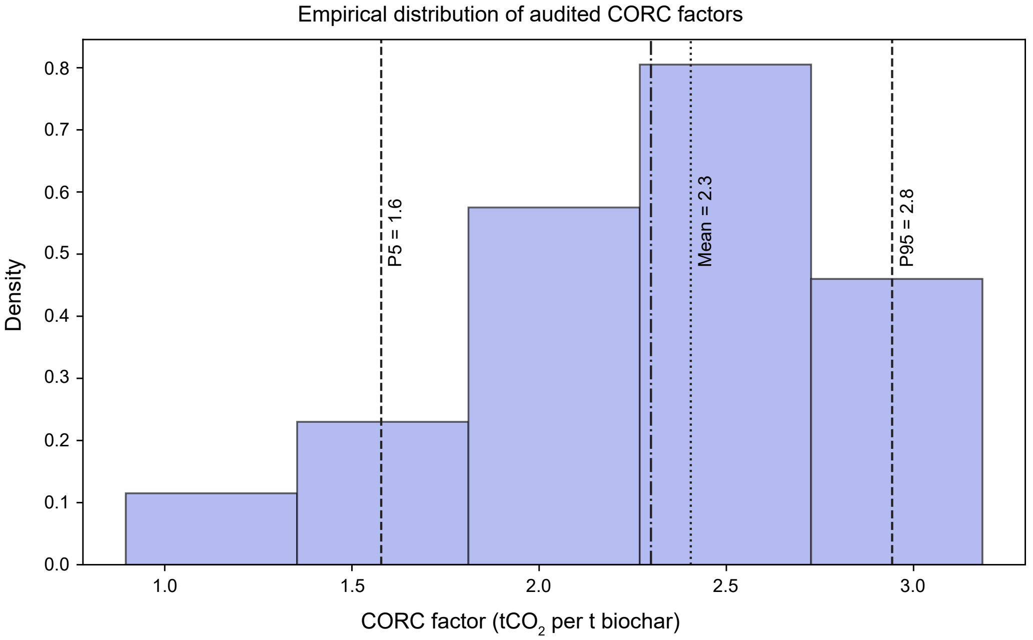

Figure S5: Empirical distribution of audited net CO_2 removal factors (CORCs, $t\,CO_2$ per t biochar; histogram). Vertical dashed lines mark the empirical 5th, median, and 95th percentiles. These 19 values are sampled directly (with replacement) in the Monte-Carlo propagation of Sections S4.

S4. Abatement-cost propagation

S4.1 Approach

To extend the capacity-specific envelopes to real-world deployment, we combine delivered-cost uncertainty from Section S3 with observed variability in carbon-removal efficiency and regional scale structure. Regional production and capacity distributions are taken from the 2023 *Global Biochar Market Report*, covering six continental regions (North America, Europe, Asia, South America, Africa, and Oceania) and four operational capacity bins (101–500, 501–1 000, 1 001–10 000, and $>$10 000 $t\,y^{-1}$), which together represent more than 99 % of installed global capacity. The 0–100 $t\,y^{-1}$ class was excluded because it lies outside the empirical fit range.

S4.2 Global biochar market characteristics

This subsection summarizes regional production patterns, feedstock preferences, and system configurations reported in the 2023 *Global Biochar Market Report*[35]. These data provide the capacity weights and qualitative context used in the Monte-Carlo propagation described below.

Table S11: Regional distribution of producers by size category (% of regional production). The 0–100 t y^{-1} class is excluded from uncertainty propagation (outside fit range) but reported here for completeness.

**Region**	**>10,000 t y^{-1}**	**1,001–10,000 t y^{-1}**	**501–1,000 t y^{-1}**	**101–500 t y^{-1}**	**0–100 t y^{-1}**	**Installed (t y^{-1})**
North America	71.0	25.0	3.0	1.0	0.0	169,564
Europe	22.0	72.0	3.0	2.0	1.0	60,549
Asia	45.0	40.0	10.0	4.0	1.0	54,353
South America	76.0	22.0	2.0	0.0	0.0	36,247
Africa	0.0	89.0	6.0	4.0	1.0	27,440
Oceania	0.0	99.0	0.0	0.1	0.9	4,153

Table S12: Regional biomass feedstock distribution (% of total feedstock use).

**Region**	**FR**	**AR**	**MW**	**M**	**PGC**	**B**	**O**
North America	45.00	18.00	19.00	5.00	4.00	4.00	5.00
Europe	47.52	18.81	19.80	3.96	3.96	2.97	2.97
Asia	22.22	34.34	13.13	12.12	13.13	2.02	3.03
South America	36.73	27.55	6.12	11.22	6.12	3.06	9.18
Africa	28.00	35.00	14.00	8.00	10.00	2.00	3.00
Oceania	58.00	17.00	17.00	0.00	0.00	0.00	8.00

Abbreviations: FR = Forestry residues; AR = Agricultural residues; MW = Municipal wastes; M = Manure; PGC = Purpose-grown crops; B = Biosolids; O = Other.

Table S13: Regional technology distribution for biochar production (% of total production).

**Region**	**SAK**	**SBR**	**HCFA**	**MC**	**PK**	**Other**
North America	73	2	5	1	0	19
Europe	27	7	0	15	16	35
Asia	32	20	0	0	40	8
South America	100	0	0	0	0	0
Africa	16	20	0	10	12	42
Oceania	100	0	0	0	0	0

Abbreviations: SAK = Stationary auger/rotary kiln; SBR = Stationary batch reactor; HCFA = High-carbon fly ash; MC = Mobile carbonizers; PK = Portable kiln.

S4.3 Delivered-cost sampling

For each representative capacity (300, 750, 5 500, and 10 000 t y^{-1}), delivered-cost draws are taken from the additive Monte-Carlo uncertainty envelopes (Section S3) using triangular sampling between the P_5, P_{50}, and P_{95} points. Within each region, capacity draws are weighted according to the regional share of each capacity bin, yielding a regional distribution of delivered minimum selling prices (MSPs; \$/t biochar).

S4.4 Empirical CORC sampling

Carbon-removal variability is represented by direct random sampling (with replacement) from the empirical array of verified CORC factors for 19 audited facilities (0.896–3.184 t CO_2 t^{-1} biochar). No statistical fitting, truncation, or biasing is applied. Each abatement-cost realization is computed as

$$A = \frac{C_{\text{delivered}}}{\text{CORC}}.$$

S4.5 Regional and global propagation

For each region, 200 000 independent Monte-Carlo realizations were generated, each pairing a delivered-cost draw with an empirical CORC draw. The resulting abatement-cost distributions are summarized by the 5th, 25th, 50th, 75th, and 95th percentiles and the mean. A global distribution was then constructed by sampling regions in proportion to their installed capacity and repeating the same propagation procedure. For visualization, the plotted density was truncated at 1000 \$/t CO_2 (this does not affect the reported statistics).

S4.6 Results

Table S14 reports regional summaries. The global capacity-weighted distribution yields

$$\mathrm{P}_5 = 111, \quad \mathrm{P}_{25} = 144, \quad \mathrm{P}_{50} = 177, \quad \mathrm{P}_{75} = 225, \quad \mathrm{P}_{95} = 497, \quad \overline{A} = 218 \quad (\text{\$/t CO}_2).$$

Figure S5 shows the global probability density function with percentile markers (truncated at 1000 \$/t CO_2). Regional medians cluster within ±15 % of the global median, with higher costs in regions dominated by smaller plants and lower costs where large-scale units prevail. The long right tail arises from coincident draws of high delivered cost at small capacities and low-efficiency CORC factors.

Table S14: Regional abatement-cost percentiles from empirical-CORC propagation (units: \$/t CO_2).

Region	P_5	P_{25}	P_{50}	P_{75}	P_{95}	Mean
North America	107	137	167	210	439	200
Europe	122	159	192	240	515	231
Asia	113	148	185	254	650	256
South America	107	136	164	204	386	187
Africa	137	171	206	264	631	266
Oceania	136	168	198	240	407	219
Global (capacity-weighted)	111	144	177	225	497	218

S4.7 Implications

The regional and global distributions presented here provide a probabilistic estimate of the effective climate-service cost of the current biochar market. Together with Section S3, they show that (i) mean abatement cost declines with production scale, while (ii) uncertainty bands remain broad but stable in relative terms. These results inform risk-adjusted procurement and policy design for durable carbon removal.

S5. Waste-to-SAF abatement cost

This section documents the construction of the waste-to–sustainable aviation fuel (SAF) dataset used to generate the abatement-cost distribution shown in the main text. Sixteen independent techno-economic assessment (TEA) cases were collected from the peer-reviewed literature and technical reports, covering four major conversion pathways: alcohol-to-jet (ATJ), hydroprocessed

esters and fatty acids (HEFA), Fischer–Tropsch (FT), and catalytic fast pyrolysis with hydrotreating (CFP). All selected cases correspond to waste- or residue-derived feedstocks, ensuring a like-for-like comparison with the waste-to-biochar systems analyzed in Sections S1–S4.

S5.1 Scope and data extraction

For each SAF pathway, we extracted (i) the minimum selling price (MSP) or levelized cost of fuel, (ii) the full life-cycle carbon intensity (CI), and (iii) the reference year of reporting. Abatement cost was then computed as the cost per tonne of CO_2 avoided relative to fossil jet fuel:

$$A = \frac{\mathrm{MSP}_{\mathrm{SAF}} - \mathrm{MSP}_{\mathrm{fossil}}}{\mathrm{CI}_{\mathrm{fossil}} - \mathrm{CI}_{\mathrm{SAF}}}$$

All MSPs were harmonized to constant 2024 USD using the U.S. CPI-U inflation index, consistent with the biochar cost-harmonization procedure described in Section S1.3.

S5.2 Harmonized abatement costs

Table S15 summarizes the resulting 2024-USD abatement costs for each SAF pathway. Costs span more than an order of magnitude, reflecting differences in conversion efficiency, hydrogen demand, scaling assumptions, and the heterogeneity of waste-based feedstocks. These sixteen values constitute the empirical dataset used to construct the SAF abatement-cost distribution in the main text.

Table S15: Harmonized 2024 abatement costs for sixteen waste-to-SAF pathways.

**Feedstock / Pathway**	**Process**	**2024 USD/tCO_2**	**Reference**
Corn stover (agri. residue)	ATJ (ETJ)	384	Uddin et al. (2025)[36]
Harvest & thinning residues	ATJ (ETJ)	938	Akter et al. (2024)[37]
Harvest & thinning residues	ATJ (Iso-BTJ)	978	Akter et al. (2024)[37]
Yellow grease (UCO)	HEFA	493	Tao et al. (2017)[38]
Waste oil (low case)	HEFA	112	ICCT (2024)[39]
Waste oil (medium case)	HEFA	231	ICCT (2024)[39]
Waste oil (high case)	HEFA	526	ICCT (2024)[39]
MSW	FT (low)	57	ICCT (2024)[39]
MSW	FT (medium)	197	ICCT (2024)[39]
MSW	FT (high)	404	ICCT (2024)[39]
Agricultural residues	FT (low)	342	ICCT (2024)[39]
Agricultural residues	FT (medium)	594	ICCT (2024)[39]
Agricultural residues	FT (high)	621	ICCT (2024)[39]
Woody residues	FT	114	Tao et al. (2022)[40]
Forest residues & woody waste	CFP (17-O)	662	Griffin et al. (2024)[41]
Forest residues & woody waste	CFP (20-O)	641	Griffin et al. (2024)[41]

S5.3 Distribution fitting and statistical treatment

The abatement-cost values in Table S15 form a right-skewed empirical distribution with pronounced spread across pathways. To enable direct comparison with the waste-to-biochar distribution developed in Sections S3–S4, the SAF data were fit to a lognormal probability density function using the empirical 5th, 50th, and 95th percentiles:

$$A \sim \mathrm{Lognormal}(\mu, \sigma) \quad \text{such that} \quad P_5,\ P_{50},\ P_{95} \text{ match the observed SAF percentiles.}$$

This approach ensures a consistent, "apples-to-apples" comparison of the two climate-service technologies under a unified probabilistic framework.

The fitted SAF distribution exhibits:

- median (P_{50}) $\approx$ 448 USD/tCO_2,
- P_5–P_{95} range $\approx$ 98–948 USD/tCO_2,

in agreement with the violin plot shown in the main text (Figure 4). The long upper tail reflects high-cost biochemical pathways (ATJ) and catalytic fast-pyrolysis systems (CFP), while the lower tail is defined by favorable FT and HEFA scenarios using highly carbon-efficient, energy-lean feedstocks such as MSW and waste oils.

S5.4 Summary

The sixteen harmonized SAF pathways summarized here represent the current techno-economic and carbon-efficiency landscape for waste-derived SAF production. When evaluated on a durability-agnostic abatement-cost basis using a consistent lognormal fitting approach, the SAF distribution occupies a substantially higher cost regime than the waste-to-biochar systems analyzed earlier. This provides the foundation for the climate-service comparison developed in the main manuscript.

References

1. Zimmer, T., Rudi, A., Glöser-Chahoud, S., and Schultmann, F. (2022). Techno-economic analysis of intermediate pyrolysis with solar drying: A chilean case study. Energies *15*, 2272. doi: 10.3390/en15062272.

2. Bahiri, I., Ibrahim, M.M., Gautam, and Zaabout, A. (2025). Mapping techno-economic prospects of biochar production through biomass waste pyrolysis pathway. Energy Convers. Manag. *327*, 119526. doi: 10.1016/j.enconman.2025.119526.

3. Sahoo, K., Upadhyay, A., Runge, T., Bergman, R., Puettmann, M., and Bilek, E. (2021). Life-cycle assessment and techno-economic analysis of biochar produced from forest residues using portable systems. Int. J. Life Cycle Assess. *26*, 189–213. doi: 10.1007/s11367-020-01830-9.

4. Nematian, M., Keske, C., and Ng'ombe, J.N. (2021). A techno-economic analysis of biochar production and the bioeconomy for orchard biomass. Waste Manag. *135*, 467–477. doi: 10.1016/j.wasman.2021.09.014.

5. Wehrenbrecht, N.J. Techno-economic assessment of biochar production in finland using straw (2024). URL: https://www.theseus.fi/handle/10024/872586 b.E. Thesis, LAB University of Applied Sciences.

6. Gamaralalage, D., Rodgers, S., Gill, A., Meredith, W., Bott, T., West, H., Alce, J., Snape, C., and McKechnie, J. (2025). Biowaste to biochar: a techno-economic and life cycle assessment of biochar production from food-waste digestate and its agricultural field application. Biochar *7*. doi: 10.1007/s42773-025-00456-0.

7. Haeldermans, T., Campion, L., Kuppens, T., Vanreppelen, K., Cuypers, A., and Schreurs, S. (2020). A comparative techno-economic assessment of biochar production from different residue streams using conventional and microwave pyrolysis. Bioresour. Technol. *318*, 124083. doi: 10.1016/j.biortech.2020.124083.

8. Garcia-Perez, M., Brady, M., and Tanzil, A.H. Biochar production in biomass power plants: Techno-economic and supply chain analyses. Tech. Rep. Washington State University, Department of Ecology (2019). URL: https://wpcdn.web.wsu.edu/cahnrs/uploads/sites/44/Biochar-Production-in-Biomass-Power-Plants-.pdf.

9. Hu, M., Guo, K., Zhou, H., Zhu, W., Deng, L., and Dai, L. (2024). Techno-economic assessment of swine manure biochar production in large-scale piggeries in china. Energy *308*, 133037. doi: 10.1016/j.energy.2024.133037.

10. Harsono, S.S., Grundmann, P., Lau, L.H., Hansen, A., Salleh, M.A.M., Meyer-Aurich, A., Idris, A., and Ghazi, T.I.M. (2013). Energy balances, greenhouse gas emissions and economics of biochar production from palm oil empty fruit bunches. Resour. Conserv. Recycl. *77*, 108–115. doi: 10.1016/j.resconrec.2013.04.005.

11. U.S. Bureau of Labor Statistics (2024). Consumer price index. https://www.bls.gov/cpi/. .

12. Eurostat (2024). Data tools. https://ec.europa.eu/eurostat/. .

13. UK Office for National Statistics (2024). Consumer price inflation. https://www.ons.gov.uk/. .

14. Internal Revenue Service (2024). Yearly average currency exchange rates. https://www.irs.gov/. .

15. Sahoo, K., Bilek, E., Bergman, R., and Mani, S. (2019). Techno-economic analysis of producing solid biofuels and biochar from forest residues using portable systems. Appl. Energy *235*, 578–590. doi: 10.1016/j.apenergy.2018.10.076.

16. Intergovernmental Panel on Climate Change (IPCC) (2019). Appendix 4: Method for estimating the change in mineral soil organic carbon stocks from biochar amendments: Basis for future methodological development. In 2019 Refinement to the 2006 IPCC Guidelines for National Greenhouse Gas Inventories. Volume 4: Agriculture, Forestry and Other Land Use (AFOLU). Geneva, Switzerland. URL: https://www.ipcc-nggip.iges.or.jp/public/2019rf/vol4.html.

17. bio.inspecta AG. Audit report: Carbon cycle gmbh & co. kg, rieden. Tech. Rep. Puro.earth Rieden, Germany (2024). Audit period: 01 Oct 2023–31 Aug 2024. Net factor: 2.916 t CO_2 t^{-1} biochar.

18. bio.inspecta AG. Audit report: Novocarbo baltic sea. Tech. Rep. Puro.earth Grevesmühlen, Germany (2024). Audit period: 11 Feb 2024–30 Jun 2024. Net factor: 2.37 t CO_2 t^{-1} biochar.

19. bio.inspecta AG. Output audit statement: Novo carbo gmbh, dörth. Tech. Rep. Puro.earth Dörth, Germany (2023). Audit period: 28 Sep 2022–10 Jun 2023. Sub-period factors: 2.310, 2.407, 2.386, 2.517 t CO_2 t^{-1}; average 2.405.

20. EnergyLink Services Pty Ltd. Final audit report: Freres engineered wood, lyons or. Tech. Rep. Puro.earth Lyons, Oregon, USA (2024). Audit period: 01 Jul 2022–31 Aug 2023. Net factor: 2.4736 t CO_2 t^{-1} biochar.

21. EnergyLink Services Pty Ltd. Production output audit statement: Exomad green, concepción. Tech. Rep. Puro.earth Concepción, Bolivia (2025). Audit period: 22 Mar 2024–24 Mar 2025. Net factor: 2.517 t CO_2 t^{-1} biochar.

22. Earthood Services Limited. Output audit report: Planboo eco ab, farm gai kaisa 159. Tech. Rep. Puro.earth Grootfontein District, Namibia (2025). Audit period: 21 Feb 2024–24 Jan 2025. Net factor: 2.06 t CO_2 t^{-1} biochar.

23. EnergyLink Services Pty Ltd. Final audit report: Bio-logical mt kenya biochar project. Tech. Rep. Puro.earth Kabati, Murang'a, Kenya (2025). Audit period: 22 Mar 2024–28 Feb 2025. Net factors: 2.231 (Macadamia), 1.841 (Prosopis) t CO_2 t^{-1}.

24. EnergyLink Services Pty Ltd. Output audit statement: Oregon biochar solutions (primary biochar). Tech. Rep. Puro.earth White City, Oregon, USA (2022). Audit period: 01 Jul 2022–30 Sep 2022. Net factor: 1.80 t CO_2 t^{-1} biochar.

25. EnergyLink Services Pty Ltd. Final audit report: Glanris, olive branch ms. Tech. Rep. Puro.earth Olive Branch, Mississippi, USA (2023). Audit period: 01 Apr 2021–31 Aug 2022. Net factor: 0.896 t CO_2 t^{-1} biochar.

26. EnergyLink Services Pty Ltd. Final audit report: Aperam bioenergia ltda. Tech. Rep. Puro.earth Capelinha, Brazil (2024). Audit period: 01 May 2023–29 Feb 2024. Net factor: 1.654 t CO_2 t^{-1} biochar.

27. EnergyLink Services Pty Ltd. Production facility and output audit statement: Pyrocal pty ltd – wellcamp. Tech. Rep. Puro.earth Wellcamp, Queensland, Australia (2024). Audit period: 01 Jan 2022–31 Jul 2023. Net factor: 1.895 t CO_2 t^{-1} biochar.

28. bio.inspecta AG. Output audit statement: Nordgau carbon gmbh & co. kg, wernberg-köblitz. Tech. Rep. Puro.earth Wernberg-Köblitz, Germany (2024). Audit period: 01 Apr 2022–31 Mar 2023. Net factor: 2.759 t CO_2 t^{-1} biochar.

29. DNV Business Assurance Finland Oy Ab. Output audit statement: Bussme biochar ab – munka-ljungby. Tech. Rep. Puro.earth Munka-Ljungby, Sweden (2025). Audit period: 01 Jan 2023–31 Jan 2024. Net factor: 2.547 t CO_2 t^{-1} biochar.

30. DNV Business Assurance Finland Oy Ab. Output audit statement: Bussme biochar ab – svedala. Tech. Rep. Puro.earth Svedala, Sweden (2025). Audit period: 01 Jan 2023–29 Feb 2024. Net factor: 2.789 t CO_2 t^{-1} biochar.

31. DNV Business Assurance Finland Oy Ab. Facility and output audit report: Carbofex oy – nokia 1. Tech. Rep. Puro.earth Nokia, Finland (2023). Audit period: 01 Sep 2022–28 Feb 2023. Net factor: 3.184 t CO_2 t^{-1} biochar.

32. bio.inspecta AG. Output audit statement: Groupe bordet, leuglay. Tech. Rep. Puro.earth Leuglay, France (2024). Audit period: 22 Sep 2022–30 Apr 2023. Net factor: 2.659 t CO_2 t^{-1} biochar.

33. RSM Australia Pty Ltd. Production facility and output audit statement: Carbonchip mobile carboniser project. Tech. Rep. Puro.earth Australia (2025). Audit period: 12 Oct 2021–20 Dec 2023. Net factor: 2.67 t CO_2 t^{-1} biochar.

34. 350Solutions, Inc. Production facility & output audit statement: Restoration fuels, john day or. Tech. Rep. Puro.earth John Day, Oregon, USA (2024). Audit period: 01 Nov 2023–31 Mar 2024. Audited CORCs: 877; dry biochar: 434.8 t; implied factor $\approx$ 2.02 t CO_2 t^{-1}.

35. International Biochar Initiative. 2023 global biochar market report. Tech. Rep. International Biochar Initiative (2024). URL: https://biochar-international.org/wp-content/uploads/2024/06/Global-Biochar-Market-Report-2023-%E2%80%93-Public.pdf.

36. Uddin, M.M., Lee, U., Xu, H., Li, Y., Kwon, H., Zhang, Y., Smolinski, S., Cai, H., and Tao, L. (2025). Sustainable aviation fuel from ethanol: Techno-economic analysis and life cycle analysis. Appl. Energy *398*, 126373. doi: 10.1016/j.apenergy.2025.126373.

37. Akter, H.A., Masum, F.H., and Dwivedi, P. (2024). Life cycle emissions and unit production cost of sustainable aviation fuel from logging residues in georgia, united states. Renew. Energy *228*, 120611. doi: 10.1016/j.renene.2024.120611.

38. Tao, L., Milbrandt, A., Zhang, Y., and Wang, W.C. (2017). Techno-economic and resource analysis of hydroprocessed renewable jet fuel. Biotechnol. Biofuels Bioprod. *10*, 261. doi: 10.1186/s13068-017-0945-3.

39. Navarrete, A., Pavlenko, N., and O'Malley, J. Saf policy scorecard: Evaluating state-level sustainable aviation fuel policies in the united states. Tech. Rep. International Council on Clean Transportation Washington, DC (2024).

40. Tao, L., Harris, K., Lee, U., and Yoo, E. Techno-economic evaluation of strategies to approach net-zero carbon sustainable aviation fuel via woody biomass gasification and fischer-tropsch synthesis. Tech. Rep. National Renewable Energy Laboratory Denver, CO (2022). URL: https://docs.nrel.gov/docs/fy22osti/82703.pdf.

41. Griffin, M.B., Iisa, K., Dutta, A., Chen, X., Wrasman, C.J., Mukarakate, C., Yung, M.M., Nimlos, M.R., Tuxworth, L., Baucherel, X., Rowland, S.M., and Habas, S.E. (2024). Opening pathways for the conversion of woody biomass into sustainable aviation fuel via catalytic fast pyrolysis and hydrotreating. Green Chem. *26*, 9768–9781. doi: 10.1039/d4gc03333g.
